# Prepartum body condition score and plane of nutrition affect the hepatic transcriptome during the transition period in grazing dairy cows

**DOI:** 10.1186/s12864-016-3191-3

**Published:** 2016-11-02

**Authors:** M. Vailati-Riboni, S. Meier, C. R. Burke, J. K. Kay, M. D. Mitchell, C. G. Walker, M. A. Crookenden, A. Heiser, S. L. Rodriguez-Zas, J. R. Roche, J. J. Loor

**Affiliations:** 1 Department of Animal Sciences, University of Illinois, Urbana, 61801 USA; 2 DairyNZ Limited, Private Bag 3221, Hamilton, 3240 New Zealand; 3 University of Queensland, Centre for Clinical Research, Royal Brisbane & Women’s Hospital Campus, Herston, QLD 4029 Australia; 4 AgResearch, Hopkirk Research Institute, Grasslands Research Centre, Palmerston North, 4442 New Zealand

**Keywords:** Peripartum, BCS, Prepartum nutrition, Liver transcriptome

## Abstract

**Background:**

A transcriptomic approach was used to evaluate potential interactions between prepartum body condition score (BCS) and feeding management in the weeks before calving on hepatic metabolism during the periparturient period.

**Methods:**

Thirty-two mid-lactation grazing dairy cows of mixed age and breed were randomly allocated to one of four treatment groups in a 2 × 2 factorial arrangement: two prepartum BCS categories [4.0 (thin, BCS4) and 5.0 (optimal, BCS5); based on a 10-point scale], and two levels of energy intake during the 3 weeks preceding calving (75 and 125 % of estimated requirements). Liver samples were obtained at −7, 7, and 28 d relative to parturition and subsequent RNA was hybridized to the Agilent 44 K Bovine (V2) Microarray chip. The Dynamic Impact Approach was used for pathway analysis, and Ingenuity Pathway Analysis was used for gene network analysis.

**Results:**

The greater number of differentially expressed genes in BCS4 cows in response to prepartum feed allowance (1071 vs 310, over the entire transition period) indicates that these animals were more responsive to prepartum nutrition management than optimally-conditioned cows. However, independent of prepartum BCS, pathway analysis revealed that prepartal feeding level had a marked effect on carbohydrate, amino acid, lipid, and glycan metabolism. Altered carbohydrate and amino acid metabolism suggest a greater and more prolonged negative energy balance postpartum in BCS5 cows overfed prepartum. This is supported by opposite effects of prepartum feeding in BCS4 compared with BCS5 cows in pathways encompassing amino acid, vitamin, and co-factor metabolism. The prepartum feed restriction ameliorates the metabolic adaptation to the onset of lactation in BCS5 cows, while detrimentally affecting BCS4 cows, which seem to better adapt when overfed. Alterations in the glycosaminoglycans synthesis pathway support this idea, indicating better hepatic health status in feed-restricted BCS5 and overfed BCS4 cows. Furthermore, IPA network analysis suggests liver damage in feed-restricted thin cows, likely due to metabolic overload.

**Conclusion:**

Overall, the data support the hypothesis that overfeeding in late-pregnancy should be limited to underconditioned cows, while cows with optimal degree of body condition should be maintained on an energy-restricted diet.

**Electronic supplementary material:**

The online version of this article (doi:10.1186/s12864-016-3191-3) contains supplementary material, which is available to authorized users.

## Background

Nutritional management of the late pregnant dairy cow has been examined as a way to improve cow DMI and health during the transition period. The aim is to counteract the negative energy balance (NEB) that dairy cows experience postpartum, and ease their transition into lactation. Traditionally, management practices provided dry cows with a high-fiber/low-energy density ration (“far-off” diet), switching to a low-fiber/higher-energy density ration for the last month of gestation (“close-up” diet). Different studies, however, have thus far demonstrated that this “close-up” approach often leads to prepartum hyperglycemia and hyperinsulinemia and greater blood non-esterified fatty acid (NEFA) concentrations (i.e., excess lipid mobilization postpartum) [1–4]. The move to “close-up” diets with greater energy density may also elicit detrimental effects on the postpartum health of the cow [5–7].

Similar outcomes in the postpartum period have been associated with the level of body condition score (BCS) at calving. Altered plasma concentrations of biomarkers of metabolic and inflammation were detected in some studies in overconditioned cows [1, 8–10] and in “thin”, underconditioned cows [10–12], indicating that both groups are at greater risk of developing metabolic or health disorders during transition. Thin cows experience more problems in a pasture-system (the present study) than in a TMR-based system, where the risk of overconditioning during the dry period is far greater [13, 14]. For these reasons, assessment of BCS prepartum can provide a qualitative evaluation of the chances for an optimal transition, which, in turn, is closely associated with optimal production and the chances for a successful lactation [13].

Despite the fact that both prepartum BCS and plane of nutrition play an important role in the metabolic response of the animal to lactation, cows are generally managed similarly prepartum. During the transition period, the high metabolic demands required for milk synthesis rely on homeorethic mechanisms in multiple tissues, all of which converge on the metabolic ability of the liver [15]. Previous studies have demonstrated the ability of “omics” approaches for evaluating the effects of calving BCS [16, 17] and feeding management [7, 18] on the metabolic activity of the liver during the transition period. Therefore, in the present study, liver transcriptome profiling through microarray technology was undertaken to investigate the hypothesis that an interaction between precalving BCS and prepartum feeding management exists, and that it can affect the hepatic adaptations to lactation in grazing dairy cows.

## Methods

### Experimental design and sample collection

Complete details of the experimental design are available elsewhere [4]. Briefly, 150 mixed breed mid-lactation grazing dairy cows were balanced for age, breed, BCS at the time of enrollment, and expected calving date. Cows were randomly allocated to one of six treatment groups (25 cows per group) in a 2 × 3 factorial arrangement: two pre-calving BCS categories (4.0 and 5.0, BCS4 and BCS5; based on a 10-point scale, where 1 is emaciated and 10 obese; [19] and three levels of energy intake during the 3 weeks preceding calving (75, 100, and 125 % of estimated requirements; [3]). The different groups were obtained by daily manipulation of pasture allowance [4].

A subset of 32 animals with the complete set of biopsies and plasma samples (8 cows in each of four treatment groups) was used for transcriptomic analysis. These were cows with prepartum BCS4 fed to meet 75 (B4F75) or 125 (B4F125) % of requirements, and cows with prepartum BCS5 fed to meet 75 (B5F75) or 125 (B5F125) % of requirements. The average cow age was 6.4 ± 2.3, 5.4 ± 2.3, 6.3 ± 2.6, and 4.8 ± 1.5 years for B4F75, B4F125, B5F75, and B5F125, respectively. Breed-wise, pure bred New Zealand Holsteins were 4, 2, 6, and 3, for B4F75, B4F125, B5F75, and B5F125, respectively, with the remaining cows in each group being a mix-bred Jersey with an average percentage of New Zealand Holstein blood of 86 ± 10, 75 ± 19, 69 ± 24, and 47 ± 31 %, for B4F75, B4F125, B5F75, and B5F125, respectively.

The intermediate groups in both BCS classes (B4F100 and B5F100) were omitted from the present analysis as feeding to the exact requirements is rarely achievable in field conditions. In practice, multiple factors (e.g., pasture management and allocation, animal competition and social interaction) cause cows being mostly overfed or underfed.

Liver tissue was harvested via percutaneous biopsy under local anesthesia at −7, 7, and 28 d relative to parturition. Tissue samples were immediately snap frozen in liquid nitrogen and stored at −80 °C until further analysis. Blood was sampled on the same days, prior to the biopsy, by coccygeal venipuncture using evacuated blood tubes containing a lithium heparin anticoagulant, and processed for plasma collection and determination of NEFA and BHBA. Assay information is reported in Additional file 1.

### RNA extraction and microarray performance

Complete information about the procedures is reported in Additional file 1. Primer sequences and qPCR performace can be found in Additional files 2 and 3, respectively. 

### Statistical analysis

Statistical analysis for transcriptomic data was performed using SAS v9.3 (SAS Institute Inc.). Data from a total of 48 microarrays were adjusted for dye and array effects (Lowess Normalization and array centering). A MIXED model with repeated measures was then fitted to the normalized log_2_-tranformed adjusted ratios using Proc MIXED, according to the following model:

$$ {Y}_{ijklm} = \mu + {T}_i + {B}_j + {F}_k + T{B}_{ij} + T{F}_{ik} + B{F}_{jk} + TB{F}_{ijk} + {C}_l + {e}_{ijklm} $$

The model for *Y*_*ijklm*_ (dye-array adjusted expression) included the overall mean (*μ*), the fixed effects of time (*T*_*i,*_ −7, 7, and 28 d), prepartum BCS (*B*_*j*_, 4 and 5), prepartum feeding management (*F*_*k*_, 75 and 125 %), and their interactions (*TB*_*ij*_, *TF*_*ik*_, *BF*_*jk*_, *TBF*_*ijk*_). Cow (*C*_*l*_) was considered an uncorrelated random effect, and *e*_*ijklm*_ represent the residual error. The raw *P*-values were adjusted for the number of genes tested using Benjamini and Hochberg’s false discovery rate (FDR; Benjamini and Hochberg, 1995) to account for multiple comparisons.

Blood data were subjected to ANOVA in SAS (v9.3) and analyzed with PROC MIXED, fitting the same statistical model as used for transcriptome data. Time, BCS, feeding, and their interactions were considered as fixed effects, while cow, nested within treatment, was the random effect. The Kenward-Roger statement was used for computing the denominator degrees of freedom, while spatial power was used as the covariance structure. Data were considered significant at *P* ≤ 0.05 using the PDIFF statement.

### Kyoto encyclopedia of genes and genomes (KEGG) pathway analysis

The dynamic impact approach (DIA) was used for KEGG pathway analysis of differentially expressed genes (DEG). The DIA calculates the overall effect (relevance of a given pathway) and flux (direction of effect), thus allowing evaluation of transcriptome profiles in a more holistic fashion. The detailed methodology of DIA is described elsewhere [19]. Briefly, the whole dataset with Entrez gene ID, fold-change (FC) (≤ −1.5 and ≥ 1.5), and raw *P*-value (≤0.01) was uploaded to DIA. For the analyses, a minimum of 30 % annotated genes on the microarray versus the whole genome [19] was selected.

### Transcription regulators and gene network analysis

Ingenuity pathway analysis (IPA) software (Qiagen) was used to analyze the upstream transcription regulators and their connections with other downstream genes that were differentially expressed. For this purpose, a list of DEG, with the same thresholds used for DIA analysis, was uploaded into IPA. However, for IPA analysis, the time effect was not included to focus on overall interaction of BCS and prepartum feeding management along the whole transition period.

### Verification of microarray results

To verify some of the key findings from the microarray and bioinformatics analysis, the expression levels of 11 genes, including eight and three DEG for the comparisons B4F125vsB4F75 and B5F125vsB5F75, respectively, were analyzed using real-time quantitative PCR. Because of the higher number of detected DEG, validation was performed only on samples obtained at 7 days postpartum. Complete information about the procedures is reported in Additional file 1, together with the list of analyzed genes and respective pathways annotated via DIA. Per each comparison a fold-change was calculated from the normalized relative mRNA abundance obtained from each gene with its own standard curve, and compared with the fold-change from the microarray analysis. A Pearson correlation using the proc CORR procedure in SAS (v9.3) was then run to establish similarity between the two analyses. Results are reported in Fig. 6.

## Results and discussion

**Fig. 1:**
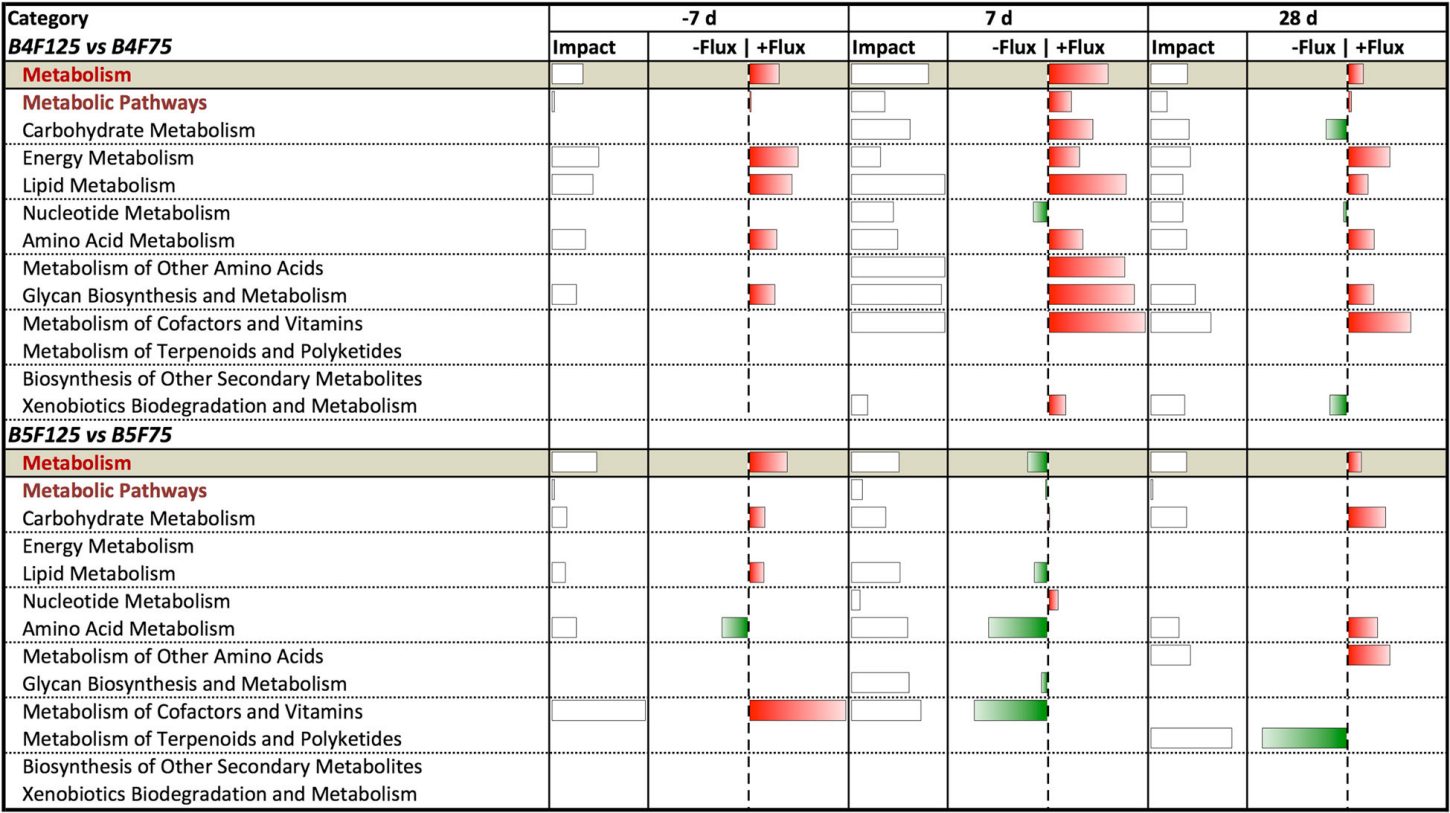
Summary of KEGG metabolic subcategories resulting from the DIA analysis in liver of BCS 4 and 5 cows fed 125 % of requirements prepartum compared with 75 %. For each sampling time, the columns represent the effect (impact) and flux responses. The transparent bars represent the effect value (0 to 30), and the flux columns represent negative (−) and positive (+) flux (−30 to +30) based on the direction of the effect. The negative flux (*green bars*) indicates an upregulation in B4F75 or B5F75 cows, while the positive flux (*red bars*) indicates an upregulation in B4F125 or B5F125 cows

**Fig. 2:**
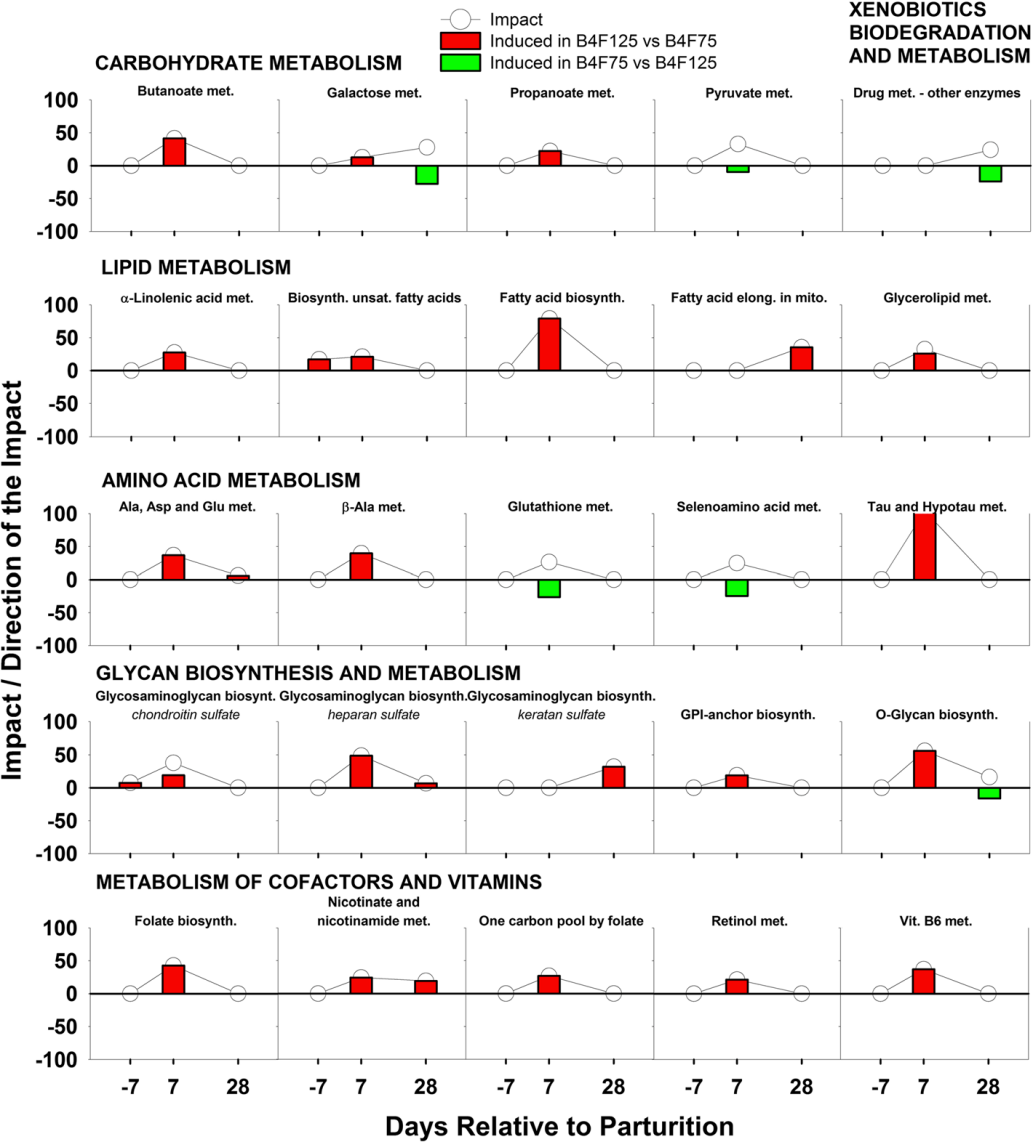
Dynamic Impact Approach (DIA) results (Impact and Direction of the Impact) for the most impacted metabolic KEGG pathways (Top 25), grouped in sub-categories of pathways, in BCS 4 cows fed 125 % compared with 75 % of requirements prepartum. *Lines* represent the Impact (0–100) along the transition period, while *bars* show the Direction of the Impact (−100–+100) (*red* = upregulation, *green* = downregulation, in B4F125 vs B4F75)

**Fig. 3:**
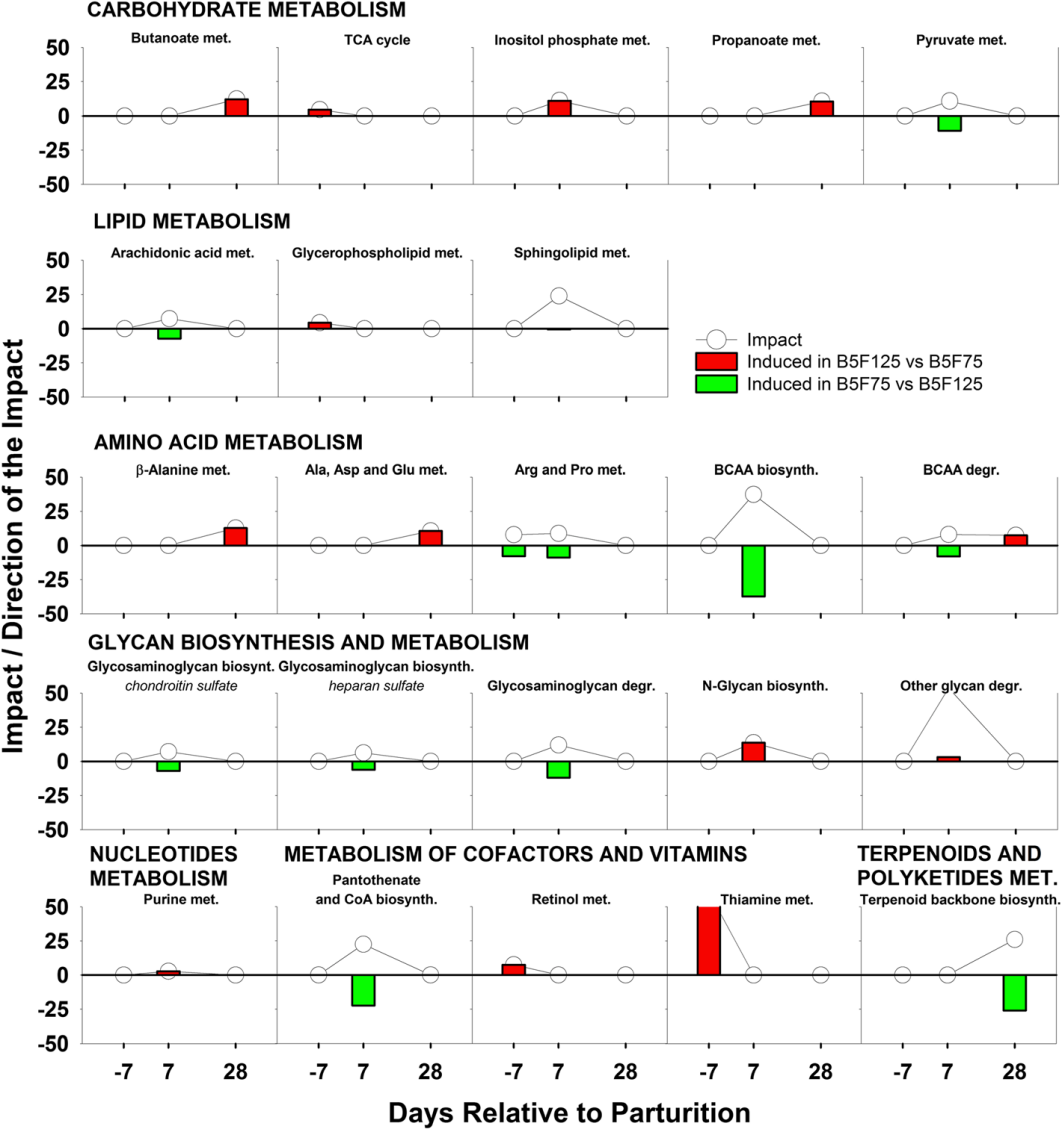
Dynamic Impact Approach (DIA) results (Impact and Direction of the Impact) for the most impacted metabolic KEGG pathways (Top 23), grouped in sub-categories of pathways, in BCS 5 cows fed 125 % compared with 75 % of requirements prepartum. Lines represent the Impact (0–50) along the transition period, while *bars* show the Direction of the Impact (−50–+50) (*red* = upregulation, *green* = downregulation, in B5F125 vs B5F75)

Despite the fact that BCS is linked with the metabolic response to lactation and its level is regulated through nutrition, cows with different levels of adiposity are generally managed similarly during the prepartum period. In light of this, the present manuscript focuses on the pre- and postpartum (−7, 7, and 28 d) metabolic effect of different prepartum feeding regimes within BCS groups (B4F125 vs B4F75, and B5F125 vs B5F75). Time was not considered in the discussion of transcription regulator and gene network analysis; instead, the focus was on the overall effect of BCS and feeding management, using the same comparisons as in the DIA analysis. Differentially expressed genes were determined as reported previously [20, 21], applying first a stringent *P* value cut-off of *P* ≤ 0.01 to the data, and then an FC threshold of ≤ −1.5 and ≥ 1.5. Validation for this approach, which does not take into account multiple testing corrections, was reported previously [22, 23]. The resulting number of DEG is reported in Table 1, while the top DEG with FC ≤/≥ ± 3 per each comparison and at each time point are reported in Additional files 4, 5, 6, 7, 8 and 9. The five main areas among the KEGG categories and subcategories that were enriched with DEG are reported in Fig. 1. Among these, the following discussion will only concern the ‘metabolism’ category, with a focus on the 25 metabolic pathways that were most-impacted by treatment (Figs. 2 and 3), emphasizing the effect of prepartum diet in combination with prepartum BCS. The term “impact” refers to the biological importance of a given pathway as a function of the change in expression of genes composing the pathway (proportion of DEG and their magnitude) in response to a treatment, condition, or change in physiological state [19]. Consequently, the direction of the impact, or flux, characterizes the average change in expression as up-regulation/activation, down-regulation/inhibition, or no change.

**Table 1:** Number of differentially expressed genes, used for DIA and IPA analysis, for each considered comparison after applying a *P* value cutoff of ≤ 0.01 and a fold change threshold at ≤/≥ ± 1.5

Comparison	Wk^a^	Up	Down	Total
*DIA*				
B4F125 vs B4F75	−7	62	63	125
	7	336	229	565
	28	147	234	381
B5F125 vs B5F75	−7	31	43	74
	7	99	106	205
	28	15	16	31
*IPA*				
B4F125 vs B4F75	n.a.	69	174	243
B5F125 vs B5F75	n.a.	56	62	118

^a^Time factor was used only for DIA analysis, while IPA analysis focused on the overall effect during the whole transition period

### The effect of body condition score

The hepatic transcriptome of BCS4 compared with BCS5 cows was more impacted by prepartum feeding management in the early postpartum (7 d), i.e. during the homeorhetic metabolic shift that the cow experiences in response to the onset of lactation. According to established management standards [13], BCS5 cows are considered optimally-conditioned at parturition, while BCS4 cows are underconditioned. Thus, the fact that the number of DEG detected due to different prepartum feeding management was on average 3x higher in BCS4 than BCS5 cows underscores how transition cow nutrition could be more important in underconditioned animals.

In both BCS4 and BCS5 cows, feeding at 125 % of requirements upregulated the prepartum metabolism-related transcriptome (Flux value in Fig. 1). However, as the change in flux indicates, immediately postpartum (7 d) BCS4 cows benefitted from the greater feed allowance (125 % vs. 75 %), because there was an overall activation of metabolic pathways. In contrast, when BCS5 cows were overfed during the dry period, the same pathways were inhibited early post-partum (Fig. 2). This indicates that a greater feed allowance prepartum might have elicited a non-desirable effect on optimally-conditioned cows, rendering them “metabolically-lazy” during the metabolically-demanding transition to lactation.

#### Carbohydrate metabolism

AW Bell [24] estimated a marked increase in demand for glucose during the transition period. Because glucose and related metabolites constitute the fulcrum of ‘Carbohydrate metabolism’ in the cow, an impact (or overall effect based on changes in gene expression) on this pathway was expected and evident right after parturition regardless of BCS (Fig. 1). However, only BCS4 cows had a greater impact (or significant change) of feeding management on ‘Carbohydrate metabolism’, with half of the subcategories impacted at 7 d (Additional file 10: Table S7).

Among the most-impacted pathways (i.e. of high biological relevance based on transcriptome), ‘Pyruvate metabolism’ was activated at 7 d postpartum when cows were fed 75 % of requirements before calving, regardless of BCS, although the timing of changes to volatile fatty acid (VFA) metabolism was BCS-dependent. Metabolism of propionate and butyrate was impacted shortly after parturition (7 d) in BCS4, and at the end of the transition period (28 d) in BCS5 cows. For both BCS groups, overfeeding prepartum led to the activation of VFA pathways. When cows were underfed prepartum in both BCS4 and 5, the high impact and flux of ‘Pyruvate metabolism’ early postpartum were due to the high expression of the cytosolic form of malic enzyme (*ME1*) (Additional file 5: Table S2 and Additional file 8: Table S5). Malic enzyme activity supplies NADPH for fatty acid biosynthesis, particularly in adipose tissue [25]; however, its role in bovine liver is unclear, as the liver is not a lipogenic organ in this species [26]. Furthermore, the idea that ‘Pyruvate metabolism’ serves to provide precursors for gluconeogenesis through oxaloacetate is inconsistent with the positive flux (activation) of the ‘Gluconeogenesis/Glycolysis’ in the BCS4 cows. In fact, data indicated an induction of this pathway in B4F125 cows. Thus, we hypothesize that, independently of BCS, the impact on ‘Pyruvate metabolism’ due to changes in *ME1* expression could be related to the more pronounced postpartum NEB in cows overfed prepartum. This is supported, at least in part, by the lower activity of liver malic enzyme during starvation in ruminants [27].

However, when interpreting the expression profile of BCS4 cows, the fact that VFA production is tightly linked with DMI (greater intake, greater VFA production) and that both ‘Propanoate metabolism’ and ‘Butanoate metabolism’ early postpartum were upregulated in B4F125 cows seems to argue against our hypothesis of a more pronounced postpartum NEB in generally overfed cows prepartum. In fact, these data (pathways of VFA metabolism, Gluconeogenesis and Fatty acid biosynthesis) oppositely indicate a greater postpartum DMI, which would lead to a less pronounced NEB in underconditioned cows when overfed rather than feed restricted during the transition period. The increase in impact of the VFA pathways in BCS5 at 28 d cows, instead, agrees with a more prolonged NEB when overfeeding prepartum [4].

#### Lipid metabolism

Despite the lower adiposity in leaner than optimally-conditioned cows (Fig. 1), ‘lipid metabolism’ was more impacted as a consequence of prepartum nutrition management in BCS4 than BCS5 cows. Furthermore, the changes in flux early postpartum indicated that feeding BCS4 cows at 125 % of requirements had an activation effect, but in BCS5 cows it had a slight inhibitory effect. Also, because plasma NEFA were overall higher in BCS5 cows compared with BCS4 (Table 2), the slight inhibition of lipid metabolism in BCS5 cows resulting from prepartum overfeeding could compromise the ability of the liver to handle mobilized fatty acids in early lactation. In contrast, despite the lower hepatic flux of NEFA, prepartum overfeeding seems to have ‘prepared’ the liver of under-conditioned cows by matching post-partum lipolysis with an up-regulation of lipid metabolism.

**Table 2:** Effect of prepartum body condition score (BCS) and feeding management on plasma concentration of NEFA and BHBA in dairy cows during the transition period

	B		F			*P-*value^1^						
mmol/L	4	5	75 %	125 %	SE	B	F	B*F	T	B*T	F*T	B*F*T
NEFA	0.50^**x**^	0.72^**y**^	0.59	0.62	0.04	0.0004	0.62	0.99	<0.0001	0.05	0.26	0.99
BHBA	0.46^**x**^	0.60^**y**^	0.57^**x**^	0.49^**y**^	0.03	0.004	0.05	0.83	<0.0001	0.10	0.01	0.57

^1^ *T* time (week relative to parturition), *B* BCS (1–10 scale), *F* level of feeding prepartum relative to requirement (%), B*F*T interaction of BCS, level of feeding prepartum relative to requirements (%), and time around parturition

x, y, z = significant difference among groups (*P* < 0.05)

Within the top impacted pathways (Figs. 2 and 3), none overlapped between the two BCS categories underscoring that prepartum nutrition management had a unique effect that was dependent on degree of adiposity. A complete discussion for each comparison of the pathways involved in the “Lipid Metabolism” category is available in Additional file 1.

#### Amino acid metabolism

Although cows mobilize less energy from muscle protein than from fat reserves, muscle protein mobilization occurs mainly in the first few weeks after calving [28, 29]. Muscle catabolism is of great importance during NEB, not only as a “provider” of amino acids for milk protein synthesis, but also of gluconeogenic precursors to the liver [30].

In both BCS groups, ‘amino acid (AA) metabolism’ was among the most-impacted categories (Fig. 1), as cows experienced an activation of the related pathways all through the transition period. However, when feeding management was considered, clear differences emerged between groups. In fact, prepartum and early postpartum, the fluxes indicated a greater activation of AA metabolism in BCS4 cows when fed 125 % of requirements, while in BCS5 cows it occurred when fed at 75 %. At 28 d postpartum, feeding at 125 % seemed to activate this pathway in both BCS groups.

Independently of BCS, the ‘Alanine, Aspartate, and Glutamate metabolism’ pathway was impacted after parturition. Because this pathway revolves around the citric acid cycle, as the three amino acids are all gluconeogenic, its link with muscle protein catabolism is logical. This is supported by published data on free amino acid concentrations in muscle (and similar trends in plasma), where Ala, Asp and Glu experience an average ~2-fold increase at 3 weeks postpartum compared with 1 week prepartum [31].

The ‘β-Alanine metabolism’ pathway is closely linked with ‘Alanine, Aspartate, and Glutamate metabolism’, because L-Asp can be converted to β-Alanine. The impact and flux of the two pathways, in fact, matched very closely (Figs. 2 and 3). β-Alanine could be used to synthesize carnosine. Among its biological activities, carnosine can scavenge reactive oxygen and nitrogen species [32]. In liver tissue it can also act as an antioxidant by increasing vitamin E and Zn-Superoxide dismutase activity [33]. Thus, a putative increase in hepatic concentration of carnosine in B5F125 cows at 28 d might be connected to a greater need of antioxidants due to a possible heightened state of oxidative stress. Unlike B5F125 cows, a possible increase of carnosine in B4F125 cows supports the hypothesis discussed in subsequent sections (B4F125 vs B4F75 -Amino acid metabolism) of a concerted activation of protective mechanisms to account for the responses in pathways related to glutathione, taurine and hypotaurine, and selenoamino acid metabolism.

#### Glycan biosynthesis and metabolism

Together with ‘Carbohydrate’ and ‘Amino acid metabolism’, this subcategory contains most of the top impacted metabolic pathways. Glycans are carbohydrates linked with lipid and protein moieties to form glycolipids and glycoproteins that can act as signaling molecules [34]. Despite previous work with dairy cows [7, 19, 35] demonstrating substantial changes in several glycan-related pathways in liver tissue during the transition period, not much attention has been placed on glycan metabolism. However, a review of the literature highlighted that there has been a marked increase in glycomic studies in humans [36], highlighting their importance. Further research will soon be required to understand the role of glycans in dairy cow liver during the transition period, as, in fact, glycan modifications of intracellular and extracellular proteins have critical functions in almost all biological pathways [37].

The most-impacted glycan pathways in our experiment included both biosynthesis [Glycosaminoglycan (GAG), Glycosylphosphatidylinositol (GPI)-anchor, O- and N-Glycan] and degradation (Glycosaminoglycan, other glycans) (Figs. 2 and 3). However, the degradation pathways were impacted in response to feeding management only in BCS5 cows. Glycosaminoglycans are the most abundant heteropolysaccharides in the body [38]. Based on core disaccharide structures, GAG are classified into heparin sulfate, chondroitin sulfate, keratin sulfate, and hyaluronan [38]. Sulfated GAG are key players in both molecular and cellular events of the regulation of inflammation [39]. Most importantly, they display anti-inflammatory functions in humans [39]. The synthesis of all three sulfated classes was affected by feeding management in both BCS4 and BCS5 cows. However, feeding at 125 % of requirements activated the synthesis pathways in BCS4 cows, while it inhibited it in BCS5 cows. Furthermore, the overfeeding approach induced the degradation of GAG in BCS5 cows.

When you consider the three classes of sulfated glycosaminoglycans, data indicated that increasing dietary allowance prepartum in BCS4 cows induced GAG synthesis throughout the transition period. Because the transition period is characterized by a systemic level of inflammation [40], from sub-acute to acute, an increased synthesis of anti-inflammatory compounds could be beneficial and help reduce any detrimental effect while maintaining its physiological homeorhetic properties. In contrast, overfeeding seemed to reduce sulfated GAG synthesis in optimally-conditioned cows (BCS 5), supporting the idea that a restricted feeding prepartum might be a better option for fatter cows.

#### Ingenuity pathway analysis

**Fig. 4:**
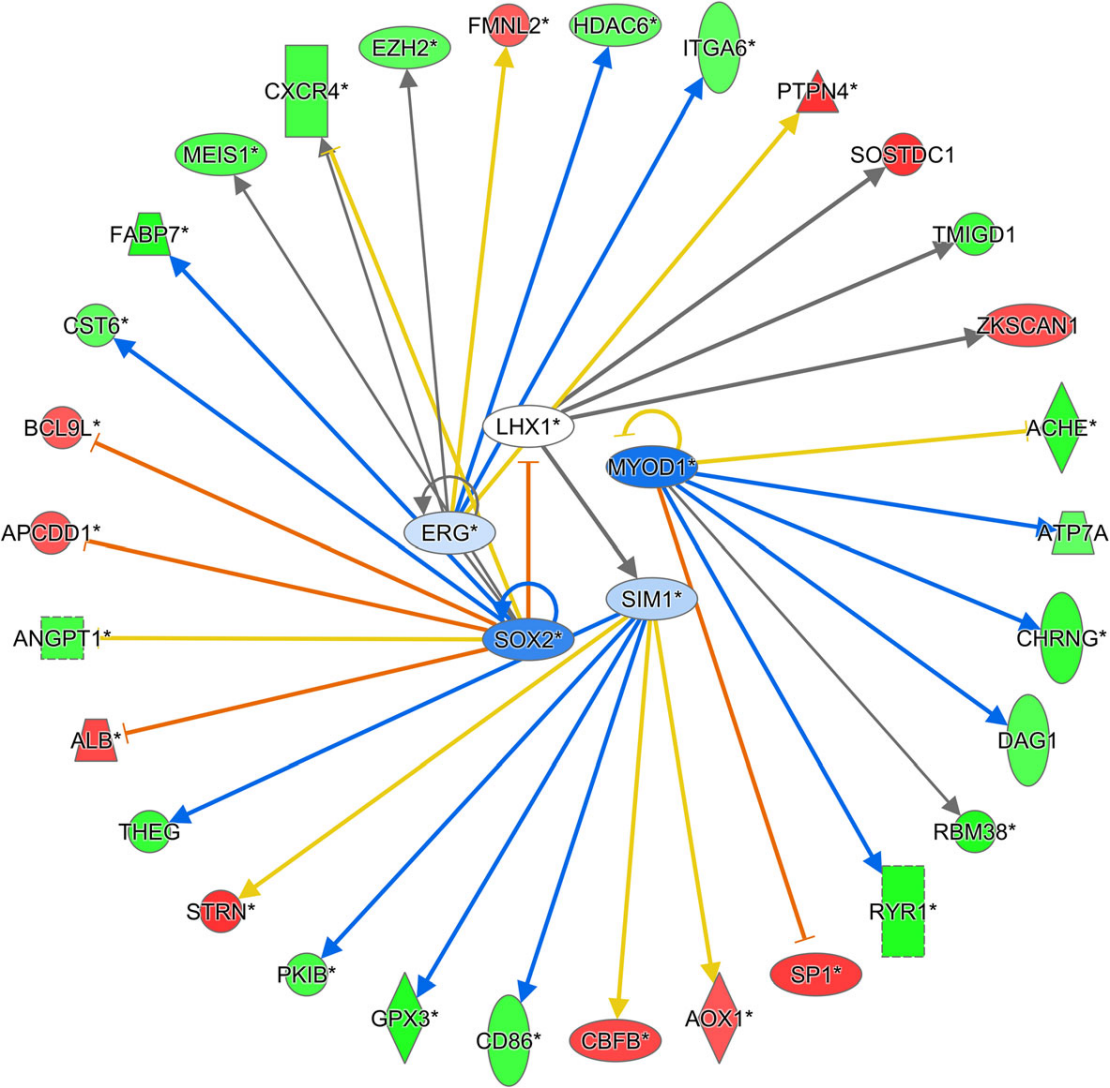
Ingenuity Pathway Analysis (IPA) upstream network analysis of differentially expressed genes in liver of B4F125 cows compared with B4F75. Upstream regulators are located at the center of the network, and the downstream target genes are located at the periphery. The *arrow* represents the direction of the target molecule. The *red* color represents upregulation of genes in B4F125, and *green* color represents upregulation of genes in B4F75. The detailed IPA legend can be found in Additional file 11: Figure S1

**Fig. 5:**
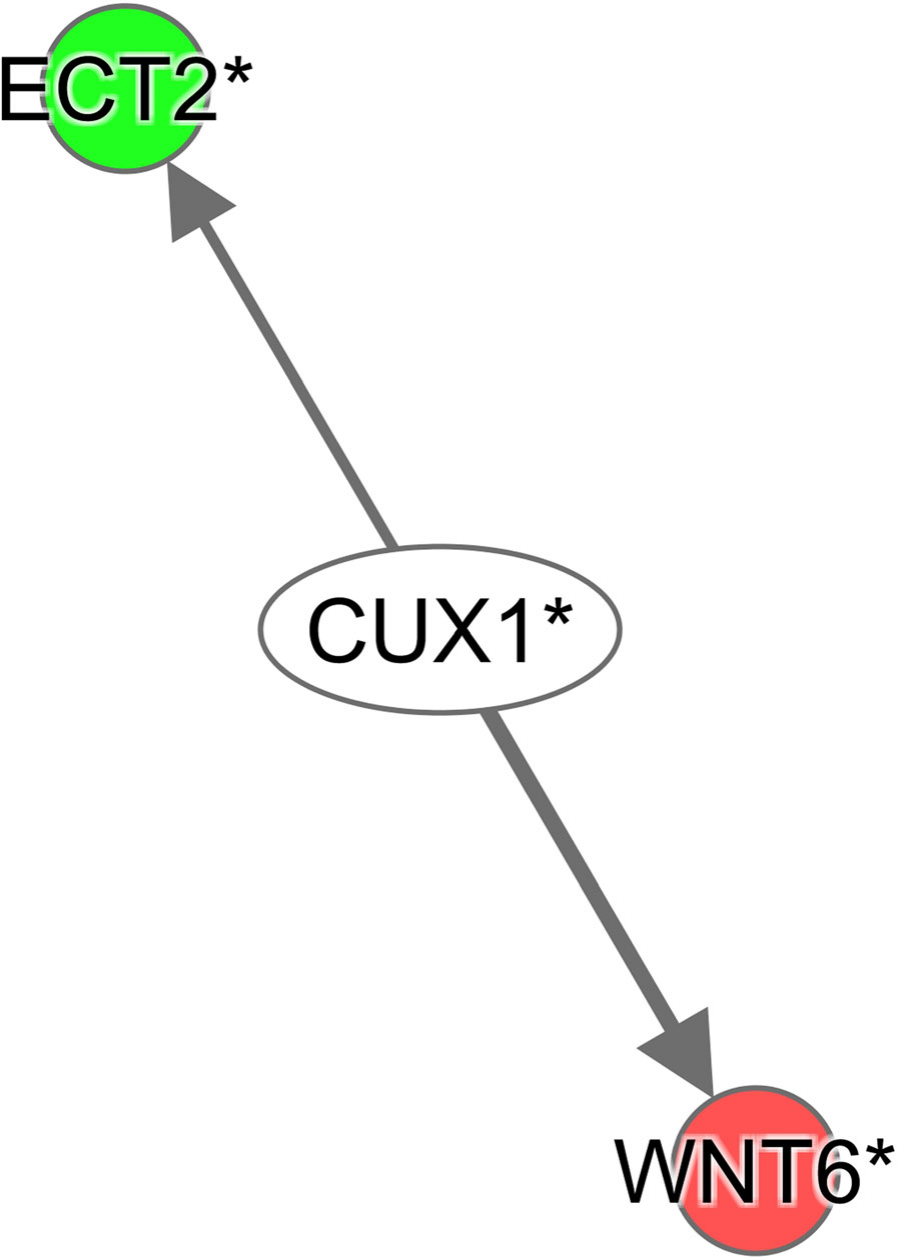
Ingenuity Pathway Analysis (IPA) upstream network analysis of differentially expressed genes in liver of B5F125 cows compared with B5F75. Upstream regulators are located at the center of the network, and the downstream target genes are located at the periphery. The *arrow* represents the direction of the target molecule. The *red* color represents upregulation of genes in B5F125, and *green* color represents upregulation of genes in B5F75. The detailed IPA legend can be found in Additional file 11: Figure S1

Using the same DIA thresholds and cutoffs comparing overfeeding with feed restriction within BCS groups, the IPA analysis identified 16 and 44 transcription regulators in BCS5 and BCS4. After considering only those with FC ≥ |1.5|, there was 1 identified transcription regulator for B5F125 compared with B5F75, and 5 for B4F125 compared with B4F75, with 2 and 32 downstream DEG affected. Prepartum overfeeding led to the downregulation of the 6 transcription factors, 5 and 1 for BCS4 and BCS5 respectively, which are somewhat connected with cell cycle and proliferation and tissue development. In BCS5 cows, the small network around *CUX1* is ambiguous as the two affected downstream genes (*ECT2* and *WNT6*) are both involved in cell proliferation and liver regeneration, but have opposite effects on the regulation of these processes. Thus, it was concluded that feeding level did not affect hepatocyte transcriptome regulators in optimally-conditioned cows. However, in BCS4 cows, probably due to the greater number of DEG, the network around the transcription regulators *ERG*, *LHX1*, *MYOD1*, *SIM1*, and *SOX2* is more consistent in its direction; hence, more defined regulatory patterns could be discerned. More detailed information about the upstream analysis is reported in Table 3, while the graphic networks are in Fig. 4 (B4F125vsB4F75) and Fig. 5 (B5F125vsB5F75). The networks's legend can be found in Additional file 11: Figure S1.

**Table 3:** Upstream differentially expressed transcription regulators and their target genes with fold change (FC) ≥ |3|

Transcription regulator	FC	Target genes and responses within the specific comparison
B4F125 vs B4F75		
*SIM1*	−1.980	*↑AOX1, ↑CBFB, ↓CD86, ↓GPX3, ↓PKIB, ↑STRN, ↓THEG*
*SOX2*	−1.766	*↑ALB, ↓ANGPT1, ↑APCDD1, ↑BCL9L, ↓CST6, ↓CXCR4, ↓FABP7, ↓MEIS1*
*MYOD1*	−1.641	*↓ACHE, ↓ATP7A, ↓CHRNG, ↓DAG1, ↓RBM38, ↓RYR1, ↑SP1*
*ERG*	−1.552	*↓CXCR4, ↓EZH2, ↑FMNL2, ↓HDAC6, ↓ITGA6, ↑ PTPN4*
*LHX1*	−1.505	*↓SIM1, ↑SOSTDC1, ↓TMIGD1, ↑ZKSCAN1*
B5F125 vs B5F75		
*CUX1*	−2.035	*↓ECT2, ↑WNT6*

### B4F125 vs B4F75

#### Carbohydrate metabolism

**Fig. 6:**
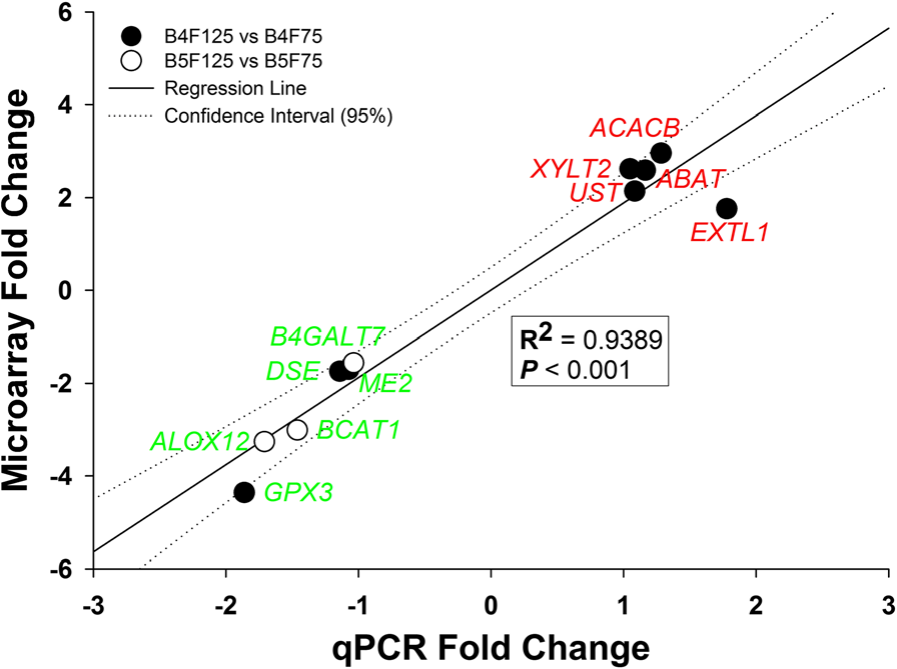
Correlation between qPCR and microarray expression fold change results. Upregulated genes are highlighted in *red*, while downregulated genes are in *green*. *Black circles* represent genes validated for the comparison B4F125vsB4F75, while *white circles* represent genes validated for the comparisons B5F125vsB5F75

‘Galactose metabolism’ was affected postpartum by feeding management, with an increase in impact from 7 to 28 d postpartum. Flux was positive (activation) at 7 d and negative (inhibition) at 28 d, underscoring an interaction between prepartum feeding and time postpartum. At 28 d *LALBA* (Lactalbumin, Alpha-) was responsible for the negative flux. Because LALBA is essential for the synthesis of lactose, the fact that lactose production takes place only in mammary cells complicates the interpretation of ‘Galactose metabolism’ and its impact on liver. However, early postpartum (7 d), *PFKL* (Phosphofructokinase) was responsible for the pathway activation. Among ‘Galactose metabolism’ related genes, *PFKL* leads to the formation of tagatose-6P, which, in rat hepatocytes has been proven to protect against pro-oxidant-induced cell injury [41]. This, again, supports the hypothesis, discussed in subsequent sections (B4F125 vs B4F75 - Amino acid metabolism), that there is an activation of protective mechanisms against oxidative damage in B4F125 cows (Fig. 6).

#### Amino acid metabolism

Unique to the BCS4 group was the activation one week after parturition of three amino acid-related pathways involved in antioxidant status (Fig. 2). ‘Glutathione metabolism’ and ‘Selenoamino acid metabolism’ were both induced in BCS4 cows fed 75 %, while ‘Taurine and Hypotaurine metabolism’ was induced when cows were fed 125 % of requirements. Among the DEG with a FC ≥ |3| (Additional file 5: Table S2), *GPX3* (Glutathione Peroxidase 3) and *MARS* (methionyl-tRNA synthetase) were the main drivers for the impact and flux of the first two pathways. Although *MARS* in the ‘Selenoamino acid metabolism’ catalyzes the incorporation of Se into proteins as seleno-Met (SeMet), this form of Met functions mainly for storage of selenium, which, once released during turnover, can be incorporated by the same pathway into seleno-Cys, a fundamental component of GPX3. Because glutathione peroxidase protects the organism from oxidative damage, we hypothesize that the induction of *GPX3* and the glutathione and selenoamino acid pathways in B4F75 cows reflects greater oxidative stress conditions postpartum when cows were feed restricted pre-calving, i.e. this management approach is likely not suitable for underconditioned cows. Even if mainly a storage form of Se, SeMet has been demonstrated to protect bovine mammary epithelial cells from H_2_O_2_-induced apoptosis and increase proliferation and cell viability under conditions of oxidative stress [42]. A similar mechanism could be present in hepatocytes.

In contrast to feeding 75 % of requirements, feeding 125 % may have strengthened hepatic antioxidant capacity by increasing the taurine and hypotaurine pool. In fact *GAD2* (glutamate decarboxylase 2; Additional file 5: Table S2) was upregulated in B4F125 cows, leading to the possible formation of the two antioxidants. By modulating gene expression, taurine has a beneficial effect on liver health, as it alleviates hepatic TAG accumulation (at least in non-ruminants). It acts by enhancing the expression of *MTTP* (microsomal triglyceride transfer protein), which improves hepatic lipid efflux. It also can enhance *PPARA* (peroxisome proliferator-activated receptor α) expression, and, in turn, increase hepatic mitochondrial and peroxisomal lipid [43]. In our dataset of affected genes, only *MTTP* was significantly up-regulated (FC = 1.3, *P* < 0.01, data not shown) by overfeeding lean cows.

The main difference between feeding management strategies was that in B4F75, the impact was due to an enzyme (*GPX3*) directly involved in the antioxidant system and production of SeMet, while in B4F125 the antioxidant pool was increased. Thus, we consider the first scenario a response to stress and the second scenario a “build up” of a defense mechanism. In conclusion, we believe that, overfeeding underconditioned cows prepartum could improve liver health postpartum at least in part by enhancing hepatic handling of tissue-mobilized fatty acids, and also by building a greater antioxidant defense against oxidative stress in the postpartum.

#### Glycan biosynthesis and metabolism

Both “O-glycans” and “GPI-anchor biosynthesis” were activated early postpartum by the prepartum increased feed allowance. O-glycans play a role in mammalian Notch signaling [44], which is important for the regulation of the complex interactions between liver cell types involved in tissue repair [45]. Because hepatocytes experience an increased work load after calving due to a shift in metabolism [15], an increase in differentiation and repair through the Notch pathway stimulated by O-glycans might be a mechanism to support high hepatocyte turnover. The fact that a persistent activation of Notch signaling is associated with liver malignancies in mouse models [45] might explain why at 28 d postpartum the impact of the pathway was reduced and the flux became slightly negative, especially in B4F125 cows.

GPI is a glycolipid that can be attached to the C-terminus of a protein during posttranslational modification, forming the so called GPI-anchored proteins [46]. In the context of the transition period, a GPI anchor is used by hepatocytes to bring the high-density lipoprotein (HDL) binding protein into contact with the blood stream [47]. HDLs contain little TAG but a large amount of cholesterol; thus, an increase in their uptake could, hypothetically, result in a greater tissue cholesterol concentration. Cholesterol is also a component of very low density lipoproteins (VLDL) and its concentration can regulate liver apolipoprotein B synthesis (ApoB), another indispensable component of VLDL, during hepatic lipid export [48]. Although *APOB* was upregulated 1.2-fold it did not reach statistical significance (*P* = 0.10), but it should be kept in mind that synthesis of APOB is primarily regulated at the post-translational level [49]. Immediately post-partum cows increase adipose tissue lipolysis [15] therefore, an increase of VLDL synthesis could help the liver of B4F125 cows repackage mobilized fat, thereby avoiding the detrimental effect of hepatic TAG accumulation.

#### Metabolism of cofactors and vitamins

The postpartum impact of ‘Retinol metabolism’ detected in B4F125 cows reinforces the idea (as previously hypothesized) that modest overfeeding in underconditioned cows in the dry period leads to better DMI and, possibly, a lower degree of NEB in early lactation because there were no differences in milk production detected [4]. The same hypothesis of a better energy status postpartum in B4F125 than B4F75 cows emerges when examining the pattern of ‘Nicotinate and nicotinamide metabolism’. Flux through ‘Nicotinate and nicotinamide metabolism’ leads to the synthesis of some of the most-important mediators of energy metabolism reactions, the nicotinamide adenine dinucleotides (NADs) family, which includes NAD^+^, NADH, and the phosphorylated versions NADP^+^ and NADPH. The sustained upregulation of ‘Nicotinate and nicotinamide metabolism’ postpartum indicates a beneficial effect of prepartum overfeeding in underconditioned cows, i.e. a greater amount of these mediators would indicate that they are better able to cope with the energetic metabolic demand of early lactation. Among them, NAD^+^ might play the most-important role, as it is necessary to maintain the hepatic fatty acid oxidative capacity [50] and also because the lipogenic capacity of ruminant liver is quite low [26]. The involvement of NADPH in the cellular antioxidant system [51] might also indicate a better oxidative status of B4F125 compared with B4F75 cows.

Alterations in ‘Folate biosynthesis’, ‘One carbon pool by folate’, and ‘Vitamin B6 metabolism’ also revealed a possible positive effect of prepartum overfeeding in underconditioned cows. It is important to note that these three pathways are connected with the Met cycle. Folate, through 5-methyl tetrahydrofolate and vitamin B6, can, in fact, donate the methyl group required by Met synthase to synthesize Met from homocysteine [52]. Furthermore, vitamin B6 is a cofactor of cystathionine β-synthase and cystathionine γ-lyase that lead to the formation of cystathionine [53], a precursor for the antioxidants glutathione, taurine, and hypotaurine. The changes detected in these three pathways seem to indicate a greater flux through the Met cycle, with a potential increase in the availability of Met and antioxidants. This hypothetical effect, through the Met cycle, also could explain the increase in activation of ‘Taurine and Hypotaurine metabolism’ in early lactation in B4F125 cows.

It is well known that Met, together with Lys, is one of the most-limiting AA in a wide range of dairy diets [54]. Methionine is typically first-limiting and its supplementation alone is able to improve not only overall lactation performance, but also improves antioxidant status and overall immune function and inflammation status in transition dairy cows [55–57]. Hence, some of the observed beneficial effects of overfeeding underconditioned cows prepartum could be related to an improved Met and antioxidant balance in early lactation.

#### Ingenuity pathway analysis

Judging by the upregulation in B4F75 cows of *ANGPT1*, *DAG1*, *HDAC6*, *MEIS1*, *RBH38*, and *RYR1* (Fig. 4), the prepartum feed restriction could have resulted in some type of liver damage, including apoptosis. This idea agrees with the induction of the ‘Apoptosis’ pathway in B4F75 cows revealed by the DIA output (Additional file 4: Table S1). In parallel, the liver of these cows seemed to experience an inflammatory state at least in part due to lower *ALB*, greater *CXCR4* and *CD86*, all coupled with the induction of the cholinergic anti-inflammatory pathway (*ACHE*, *CHRNG*) and together with upregulation of genes associated with oxidative stress (*ATP7A*, *GPX3*, *TMIGD1*). In contrast, as judged by the upregulation of genes involved in growth, proliferation, and differentiation (*BCL9L*, *FMNL2*, *PTPN4*, *SP1*, *ZKSCAN1*) in B4F125 cows, the overfeeding of underconditioned cows prepartum appeared to result in a healthier liver.

To some extent the compromised state of the liver in B4F75 cows is further evidenced from results of the IPA network analysis. The downregulation of *ALB* and *FABP7* suggests a lower ability to handle the mobilized fatty acids during the transition to lactation, perhaps enhancing the susceptibility of the liver to accumulate lipid at times of high NEFA concentration in the circulation (e.g. early postpartum). The upregulation of *PKIB* (CAMP-Dependent Protein Kinase Inhibitor Beta), a potential inhibitor of protein kinase A (PKA) [58], is also noteworthy, because PKA is a direct activator of the gluconeogenic pathway in non-ruminant liver [59, 60]. Thus, the greater expression of *PKIB* in B4F75 cows could have impaired hepatic gluconeogenesis. In contrast, the lower expression of *PKIB* in B4F125 cows would have helped increase the intracellular glucose pool.

### B5F125 vs B5F75

#### Carbohydrate metabolism

Overfeeding cows in the prepartum period had an effect on ‘TCA cycle’ and ‘Inositol phosphate metabolism’. It is plausible that the activation of the TCA cycle prepartum was a result of the higher dietary energy supply; in comparison, however, the activation early postpartum of ‘Inositol phosphate metabolism’ might have been related with an inflammatory state induced by prepartum overfeeding. In fact, this pathway was primarily affected by the upregulation of *IPPK* (inositol-pentakisphosphate 2-kinase) in B5F125 compared with B5F75 cows. The IPPK protein catalyzes the formation of inositol hexakisphosphate (InsP6), a ubiquitous and abundant cytosolic inositiol phosphate. In humans, InsP6 primes and stimulates neutrophil respiratory burst [61, 62]; thus, we speculate that this metabolite could participate in the inflammatory response. However, both in vitro and in vivo, Insp6 inhibits L- and P-selectin function [63]. Thus, if InsP6 produced by the liver is secreted into blood it could impair neutrophil infiltration by downregulating the attachment molecules, thus, leading to reduced neutrophil functions when optimally-conditioned cows are overfed prepartum.

#### Amino acid metabolism

Contrary to BCS4, in BCS5 cows the ‘Alanine, Aspartate, and Glutamate metabolism’ pathway was impacted only at the end of the transition period (28 d), and was activated by overfeeding (B5F125). Because BCS5 cows calved with better body condition than BCS4, they would have been better able to cope with the NEB by drawing from the more abundant lipid reserves, as indicated by the higher NEFA concentrations (Table 2). The late activation of this amino acid pathway, as hypothesized when discussing VFA metabolism (The effect of body condition - Carbohydrate metabolism), might have been due to a prolonged postpartum NEB in the overfed BCS5 cows, which could lead to mobilization of muscle protein. This hypothesis is supported by data indicating that overconditioned cows have a more pronounced drop in DMI after calving and a more intense NEB compared with cows fed to or slightly below requirements during the dry period [4, 64, 65].

Among the most-impacted metabolic pathways, the metabolism of Arg and Pro and of the three branched-chained amino acids (BCAA), valine, leucine, and isoleucine were highly impacted (Fig. 3). The impact and flux of the’Arginine and Proline metabolism’ pathway prepartum was caused by the differential expression (FC = −3) of *CKMT1B* (Creatine kinase, mitochondrial 1B), which encodes an enzyme responsible for the formation of phosphocreatine (PCr). In normal conditions PCr is then transported to the muscle as energy storage to facilitate a quick muscle response [66]. Because the putative increase of PCr appears to have occurred prepartum in B5F75 cows, feed restriction in optimally-conditioned cows seems to position the cow in a better energy state compared with overfeeding, i.e. the liver would have extra energy to store as PCr in muscle. Furthermore, it can be hypothesized that a local increase of PCr would be useful to the liver as an “energy buffer” in the metabolically-demanding postpartum period. Also, the ‘Arginine and Proline Pathway’ was induced early postpartum in B5F75 cows compared with B5F125. Both amino acids are gluconeogenic; thus, it seems that prepartum feed-restricted optimally-conditioned cows were in a better energy state compared with overfed cows early in the postpartum. The same pathway is also used to produce putrescine, a polyamine with growth factor-like functions on liver regeneration and DNA synthesis [67]. An increase in its concentration would promote a faster turnover of hepatocytes under the metabolic load of early lactation, possibly resulting in a more active and responsive liver.

The BCAA metabolism pathway was impacted due to differences in the prepartum diet. The main affected gene (FC < −3, Additional file 8: Table S5) for both the catabolic and anabolic branches of the BCAA pathway at 7 d postpartum was *BCAT1* (Branched Chain Amino-Acid Transaminase 1, Cytosolic), which can catalyze both the deamination and amination of BCAA and related keto-acids. Recently, a higher postpartum plasma concentration of BCAA (specifically Val and Iso) in TMR-fed dairy cows was linked to greater liver function and a possible better responsiveness of the immune system [68]. In the present study, plasma AA data were not collected, but it is plausible that an increase in hepatic BCAA biosynthesis in B5F75 cows led to similar results, indicating how feed restriction would be more appropriate for optimally-conditioned cows. Furthermore, the fact that the’BCAA degradation’ pathway was induced at 28 d in B5F125 cows reflects a prolonged NEB in overfed optimally-conditioned cows, which induced protein mobilization, supporting, again, our claim for prepartum feed restriction in BCS5 cow.

#### Glycan biosynthesis and metabolism

Feeding BCS5 cows at 125 % of requirements prepartum induced an activation of the’N-glycan biosynthesis’ and’Other glycan degradation’ pathways early postpartum. A proposed role of N-Glycan biosynthesis in the liver is to handle the misfolded proteins in the endoplasmic reticulum (ER) during periods of stress [69]. As the ‘Protein processing in the endoplasmic reticulum’ pathway followed the same pattern as N-glycan biosynthesis (Additional file 10: Table S7), this might be indicative of a greater degree of ER stress in optimally-conditioned cows fed to 125 % of requirements prepartum. Because a similar induction of ER stress was also previously reported as a consequence of prepartum overfeeding [7, 65], our data further indicate that a slight feed-restriction might be a better approach for these cows.

The ’Other glycan degradation’ pathway was highly-impacted early postpartum due to a relatively small activation in B5F125 versus B5F75 cows. This pathway concerns the degradation of N-glycans and glycosphingolipids. Because pathways concerning glycosphingolipid biosynthesis were not affected (Additional file 10: Table S7), it is assumed that their liver concentration, and not that of N-glycans, which had a positive biosynthesis, decreased. These changes might be biologically relevant in transition cows, because mice fed a high-fat diet, in which glycan synthesis was suppressed [70, 71], had a decrease in liver accumulation of triacylglycerol along with a reduced hepatocyte expression of several genes associated with steatosis; this included those involved in lipogenesis, gluconeogenesis, and inflammation. Studies from different research groups have demonstrated that prepartum over-allowance of energy results in prepartum hyperglycemia and hyperinsulinemia and marked postpartum adipose tissue mobilization (i.e., greater blood NEFA concentration) [1–4, 65, 72]. Thus, the reduction in hepatic levels of glycosphingolipids early postpartum could be a mechanism to counteract the development of lipidosis in B5F125 cows due to the possible accumulation of lipid in the liver.

#### Metabolism of cofactors and vitamins

The physiological levels of retinol (vitamin A) and its precursor β-carotene are tightly correlated with dietary intake [73]. Similarly, thiamine (vitamin B1) production depends on the dietary availability of cobalt (Co), and on ruminal microbial activity and microbial biomass, which are connected to DMI. Thus, because F125 cows were allowed a greater availability of pasture, which is naturally rich in β-carotene and a possible source of Co, the activation of’Retinol metabolism’ and ‘Thiamine metabolism’ in B5F125 cows prepartum could be related to the greater pasture allowance owing to the experimental design.

The postpartum inhibition of ’Pantothenate and CoA biosynthesis’ seems to indicate that the feed restriction in the late dry period could enhance the energy status of optimally-conditioned cows (BCS5). Panthothenate is synthetized in the body as a component of Coenzyme A (CoA), an indispensable cofactor in synthesis and oxidation of fatty acids [74]. Furthermore, CoA is a component of acetyl-CoA, the crossover molecule in cellular metabolism [75]. With an apparent increase in production of CoA, B5F75 cows would have been able to better handle the mobilized adipose fatty acids, and more effectively coordinate the metabolic shift that occurs at calving.

## Conclusions

As hypothesized, the effect of prepartum plane of nutrition on hepatic function was dependent on the BCS of the cow, underscoring how these management tools need to be evaluated together to optimize the biological adaptations of the cow during the peripartum period. The more pronounced transcriptome changes in underconditioned cows highlighted that they are more sensitive to prepartum feeding level/allocation than optimally-conditioned cows. Similarly, the bioinformatics analysis revealed transcriptome signatures that indicate a greater and potentially more prolonged NEB in overfed optimally-conditioned cows and also in feed-restricted underconditioned cows. Based on gene network analysis, the latter group might be more prone to liver dysfunction. The data indicate a less pronounced mobilization and better handling of NEFA in overfed underconditioned cows. Overall, results indicate that overfeeding in late-pregnancy should be limited to underconditioned cows, while cows with optimal degree of body condition and those with greater than optimal condition should be maintained on a restricted energy diet.

## Additional files

Additional file 1: Provides detailed and complete information about the protocol used during the microarray and qPCR procedures, and blood collection and analysis. Furthermore, it provides additional discussion points, omitted from the main body of the manuscript (Additional file 8: Table S5, Additional file 2: Table S8 and Additional file 3: Table S9). (DOCX 47 kb)
Additional file 2: Table S8. Accession number, gene symbol and EntrezID, forward and reverse primer sequences and product length of target genes for microarray validation. (DOCX 86 kb)
Additional file 3: Table S9. qPCR performance of genes measured for microarray verification. (DOCX 59 kb)
Additional file 4: Table S1. Differentially expressed genes at −7 days from parturition with fold change (FC) ≤ −3 or ≥ +3 in liver of animals with BCS 4 fed either 125 (B4F125) compared with 75 (B4F75) % of requirement for the last 3 weeks before parturition. (DOCX 99 kb)
Additional file 5: Table S2. Differentially expressed genes at +7 days from parturition with fold change (FC) ≤ −3 or ≥ +3 in liver of animals with BCS 4 fed either 125 (B4F125) compared with 75 (B4F75) % of requirement for the three weeks before parturition. (DOCX 114 kb)
Additional file 6: Table S3. Differentially expressed genes at +28 days from parturition with fold change (FC) ≤ −3 or ≥ +3 in liver of animals with BCS 4 fed either 125 (B4F125) compared with 75 (B4F75) % of requirement for the three weeks before parturition. (DOCX 110 kb)
Additional file 7: Table S4. Differentially expressed genes at −7 days from parturition with fold change (FC) ≤ −3 or ≥ +3 in liver of animals with BCS 5 fed either 125 (B5F125) compared with 75 (B5F75) % of requirement for the three weeks before parturition. (DOCX 77 kb)
Additional file 8: Table S5. Differentially expressed genes at +7 days from parturition with fold change (FC) ≤ −3 or ≥ +3 in liver of animals with BCS 5 fed either 125 (B5F125) compared with 75 (B5F75) % of requirement for the three weeks before parturition. (DOCX 105 kb)
Additional file 9: Table S6. Differentially expressed genes at +28 days from parturition with fold change (FC) ≤ −3 or ≥ +3 in liver of animals with BCS 5 fed either 125 (B5F125) compared with 75 (B5F75) % of requirement for the three weeks before parturition. (DOCX 70 kb)
Additional file 10: Table S7. Complete result datasets generated by the DIA analysis. The KEGG pathways are sorted by category and subcategory. Blue bars represent the Impact along the transition period, while red and green bars depict the Direction of the Impact (red = upregulation, green = downregulation). (XLSX 77 kb)
Additional file 11: Figure S1. Ingenuity pathway analysis legend for network analysis. (PNG 65 kb)

## Abbreviations

AA: Amino acids
ACHE: Acetylcholinesterase
ALB: Albumin
ANGPT1: Angiopoietin 1
APOB: Apolipoprotein B
ATP7A: ATPase copper transporting alpha
BCAA: Branched chain amino acid
BCAT1: Branched chain amino acid transaminase 1
BCL9L: B-Cell CLL/Lymphoma 9-like
BCS: Body condition score
BHBA: Beta-hydroxybutyric acid
CD86: CD86 antigen, B-lymphocyte antigen B7-2
CHRNG: Cholinergic receptor nicotinic gamma subunit
CKMT1B: Creatine kinase, mitochondrial 1B
CoA: Coenzyme A
CUX1: Cut like homeobox 1
CXCR4: C-X-C motif chemokine receptor 4
DAG1: Dystroglycan 1
DEG: Differentially expressed gene
DIA: Dynamic impact approach
DMI: Dry matter intake
ECT2: Epithelial cell transforming 2
ER: Endoplasmatic reticulum
ERG: Erythroblast transformation-specific transcription factor ERG variant 10
FABP7: Fatty acid binding protein 7
FC: Fold change
FMNL2: Formin like 2
GAG: Glycosaminoglycan
GPI: Glycosylphosphatidylinositol
GPX3: Glutathione peroxidase 3
HDAC6: Histone deacetylase 6
HDL: High density lipoprotein
Insp6: Inositol hexakisphosphate
IPA: Ingenuity pathway analysis
KEGG: Kyoto encyclopedia of genes and genome
LALBA: Lactalbumin alpha
LHX1: LIM homeobox 1
MARS: Methionyl-TRNA synthetase
ME1: Malic enzyme 1
MEIS1: Meis homeobox 1
MTTP: Microsomal triglyceride transfer protein
MYOD1: Myogenic differentiation 1
NAD: Nicotinamide adenine dinucleotide
NEB: Negative energy balance
NEFA: Non esterified fatty acids
PCr: Phosphocreatine
PFKL: Phosphofructokinase, liver type
PKA: Protein kinase A
PKIB: CAMP-dependent protein kinase inhibitor beta
PPARA: Peroxisome proliferator activated receptor alpha
PTPN4: Protein tyrosine phosphatase, non-receptor type 4
RBM38: RNA binding motif protein 38
RYR1: Ryanodine receptor 1
SeMet: Seleno-methionine
SIM1: Single-minded family BHLH transcription factor 1
SOX2: SRY-box 2
SP1: Sp1 transcription factor
TAG: Triacylglycerol
TMIGD1: Transmembrane and immunoglobulin domain containing 1
TMR: Total mixed ratio
VFA: Volatile fatty acid
VLDL: Very low density lipoprotein
WNT6: Wnt family member 6
ZKSCAN1: Zinc finger with KRAB and SCAN domains 1
