# Supplementary material for: Prepartum body condition score and plane of nutrition affect the hepatic transcriptome during the transition period in grazing dairy cows
Source: BMC Genomics. 2016 Nov 2;17:854. doi: 10.1186/s12864-016-3191-3 (PMC5093966; doi:10.1186/s12864-016-3191-3)
Supplement: Additional file 1: — Provides detailed and complete information about the protocol used during the microarray and qPCR procedures, and blood collection and analysis. Furthermore, it provides additional discussion points, omitted from the main body of the manuscript (Additional file 8: Table S5, Additional file 2: Table S8 and Additional file 3: Table S9). (DOCX 47 kb) [file 12864_2016_3191_MOESM1_ESM.docx]

**Additional file 1**

**ADDITIONAL MATERIAL AND METHODS**

**RNA extraction**

Liver tissue (30-50 mg) was homogenized in a TissueLyser II (Qiagen GmbH) for 2 × 2 min bursts at 30 Htz. Total RNA and DNA were extracted using a Qiagen AllPrep DNA/RNA mini kit (Qiagen GmbH) as per manufacturer’s instructions and RNA was treated with DNase (Ambion DNA-free kit; Ambion Inc., Austin, TX). The RNA quantity, purity, and integrity were determined using a NanoDrop ND-1000 (NanoDrop Technologies Inc., Wilmington, DE) and the Agilent 2100 Bioanalyzer (Agilent Technologies Inc., Palo Alto, CA). Average RNA Integrity Number (**RIN**) for samples at -7, 7, and 28 d relative to parturition were 8.18 (± 0.39), 7.77 (± 0.43), and 8.17 (± 0.45), respectively. RNA samples were stored at -80 °C until analysis.

**Microarrays**

***cRNA Synthesis, Labeling, and Purification.*** The Agilent platform was chosen to conduct the microarray experiment, using the 44K Bovine (V2) gene expression microarray chip (Agilent Technologies Inc.), following the manufacturer’s protocols. Briefly, a total of 200 ng of RNA per sample were used to generate first-strand cDNA, which was reverse transcribed to cRNA using the low-input quick amp labeling kit (Agilent Technologies Inc). The resulting cRNA was labeled with either Cy3 or Cy5 fluorescent dye, purified using RNeasy mini spin columns (Qiagen), and subsequently eluted in 30 μL of DNase-RNase-free water. The NanoDrop ND-1000 (Thermo Fisher Scientific Inc., Waltham, MA) was used to confirm the manufacturer’s recommended criteria for yield and specific activity of at least 0.825 μg and ≥6.

***Hybridization and Scanning.*** The labeled cRNA was fragmented and then hybridized to the microarray slide according to manufacturer’s protocol. Briefly, 825 ng of Cy3 and Cy5 labeled cRNA sample were combined, mixed with 11 μL of 10× Blocking Agent (Agilent Technologies Inc.), 2.2 μL of 25× Fragmentation Buffer (Agilent Technologies Inc.), and nuclease-free water (to a final volume of 55 μL); and then fragmented at 60°C for 30 s. The reaction was then stopped by adding 55 μL of 2× GEx Hybridization Buffer (Agilent Technologies Inc.), and the samples were loaded onto the slide. These were hybridized in a rotating hybridization oven (Agilent Technologies Inc.) at 65°C for 17 h. The slides were washed according to the manufacturer’s recommended procedures and scanned using a GenePix 4000B scanner (Axon Instruments Inc., Sunnyvale, CA) and GenePix Pro v.6.1 software. Resulting spots where features were substandard were flagged as bad and excluded from subsequent analysis.

**qPCR Validation**

***cDNA synthesis.*** A portion of the RNA was diluted to 100 ng/μL with DNase/RNase-free water for cDNA synthesis through RT-PCR. Per each sample, 4 μL of diluted RNA, 5 μL of Random Primers (3 μg/μL; cat#11034731001, Roche) and 45 μL of DNase/RNase-free water. The mixture was incubated at 65°C for 5 min and kept on ice for 3 min. A total of 36 μL of master mix, composed of 20 μL of 5X First-Strand Buffer (cat#EP0441, Thermo Scientific), 5 μL of Oligo dT18 (Custom DNA Oligo Tubes, Integrated DNA Technologies), 10 μL of 10mM dNTP mix (cat#18427-088, Invitrogen), 1.25 μL of Reverse Transcriptase (200 U/μL as final concentration, cat#EP0441, Thermo Scientific), 0.625 μL of Rnase inhibitor (200 U/μL as final concentration, cat#EO0381, Thermo Scientific), and 8.125 μL of DNase/RNase-free water, was added. The reaction was performed in an Eppendorf Mastercycler Gradient following the appropriate temperature program: 25°C for 5 min, 42°C for 60 min, and 70°C for 5 min. The cDNA was then diluted 1:4 with DNase/RNase-free water, prior to quantitative PCR analysis.

***Real Time qPCR.*** Primer couples were designed using NCBI Primer-BLAST toll, and tested through normal PCR, using the same thermo cycle as the final qPCR analysis, and gel electrophoresis to certify the presence of a single PCR product of the expected size. The product was then purified and send for sequencing at the UIUC Core Sequencing Facility, to asses amplification of the correct target. Complete details on primer couples and amplification products sequences can be found in Additional Table 8. Quantitative PCR was performed using 4 μL diluted cDNA combined with 6 μL of a mixture composed of 5 μL SYBR Green master mix (cat#95073-05K, Quanta BioSciences), 0.4 μL each of 10 μM forward and reverse primers, and 0.2 μL DNase/RNase free water in a MicroAmp™ Optical 384-Well Reaction Plate (Applied Biosystems). Each sample was run in triplicate and a 7 point relative standard curve plus the non-template control were used. The reactions were performed in an ABI Prism 7900 HT SDS instrument using the following conditions: 2 min at 50 °C, 10 min at 95 °C, 40 cycles of 15 s at 95 °C (denaturation), and 1 min at 60 °C (annealing + extension). The presence of a single PCR product was verified by the dissociation protocol using incremental temperatures to 95 °C for 15 s plus 65 °C for 15 s. Data were calculated with the 7900 HT Sequence Detection Systems Software. The final data were normalized using the geometric mean of three internal control genes (ICG): *GAPDH*, *RPS9*, and *UXT*. Details on qPCR performances can be found in Additional Table 9.

**Blood Collection and analysis**

Blood was sampled by coccygeal venipuncture using evacuated blood tubes containing a lithium heparin anticoagulant. Samples were placed immediately on ice and centrifuged within 30 min at 1,500 × *g* for 12 min at 4°C. Following centrifugation, aspirated plasma was stored at −20°C until assayed.

Blood NEFA and BHBA analysis was performed by Gribbles Veterinary Pathology Ltd. Blood metabolites were assayed using colorimetric techniques at 37°C with a Hitachi Modular P800 analyzer (Roche Diagnostics, Indianapolis, IN). Plasma NEFA concentration (mmol/L) was measured using Wako Chemicals (Osaka, Japan) kit NEFA HR2 measuring oxidative condensation of 3-methyl-*N*-ethyl-*N*-β hydroxyethyl aniline with 4-aminoantipyrine, while plasma BHBA (mmol/L) concentrations was assessed using Roche reagent kits measuring the reduction of NAD to NADH during oxidation of d-3-hydroxybutyrate to acetoacetate.

**ADDITIONAL DISCUSSION**

**B4F125 vs B4F75**

***Lipid metabolism****.* Lipid metabolism in thinner cows was much more impacted by prepartum feeding management, with a distinct postpartum activation of every pathway among the top 25 impacted when animals were fed 125% compared with 75% of requirements. Biosynthetic pathways rather than catabolic ones were impacted around parturition. In fact, out of the five most-impacted pathways in this category, three are concerned with the biosynthesis of fatty acids, unsaturated fatty acids, and their elongation in the mitochondria. Despite the lack of change in blood NEFA (Table 2), this response could be taken as an indication of lower postpartum mobilization of body reserves in BCS4 animals fed 125% than 75%. This idea agrees with the fact that overfeeding energy during the dry period also increases the concentration of plasma insulin, hence, preventing excessive adipose tissue lipolysis [1]. We further speculate that a possible greater postpartum mobilization in B4F75 cows could increase the hepatic uptake of fatty acids, consequently inhibiting the biosynthetic pathways, which is a well-established effect in non-ruminant liver. Additionally, NEFA values are often quite variable, especially as a function of meal patterns [2, 3]. Hepatic transcriptome alterations, in comparison, reflect longer-term regulatory responses to changes in BCS and nutrition.

Among the non-biosynthetic pathways, ‘Glycerolipid metabolism’ was impacted and activated at 7 d postpartum in cows fed 125% of requirements. Because this pathway is involved in the synthesis of triacylglycerol (**TAG**) from free fatty acids, its activation could be a consequence of an increased flux of NEFA or could suggest a better ‘handling’ of the hepatic NEFA flux. Among the top DEG, the expression of *DGAT1* (diacylglycerol O-acyltransferase 1) had a fold change of + 4.5 in B4F125 cows. As the protein product of this gene catalyzes the final step of TAG synthesis, and the fact that BCS4 cows had an overall lower plasma concentration of NEFA (Table 2), the greater *DGAT1* expression indicates a faster handling of mobilized fatty acids, avoiding accumulation in the liver that could lead to partial oxidation and ketogenesis.

Also of great interest, is the activation of the ‘α-Linolenic acid metabolism’ in the first week after parturition combined with activation of the ‘Biosynthesis of unsaturated fatty acids’ in lean animals fed 125%. The α-linolenic fatty acid (C18:3) is part of both pathways, being partially oxidized for energy in the first or converted to eicosapentaenoic acid (**EPA**) and docosahexaenoic acid (**DHA**) in the second. These two ω-3 fatty acids elicit numerous effects on functional responses of cells involved in inflammation and immunity [4], all of which have been interpreted in the context of reducing inflammation, i.e. they are beneficial during inflammatory conditions [5]. The concurrent increase in activity of the two pathways leads us to hypothesize a greater availability of C18:3 at the hepatic level (because its metabolism was increased), for synthesis of EPA or DHA and, hence, an improvement of the immunological status of the cow. This scenario would be beneficial as cows always experience an inflammatory state after parturition that can impair production and reproduction, and predispose to disease [6].

**B5F125 vs B5F75**

***Lipid metabolism***. The pathways of ‘Lipid metabolism’ impacted in optimally-conditioned cows (BCS5) mainly concerned pathways of utilization rather than synthesis (as detected in the BCS4 group), and they were mostly impacted in early lactation (7 d). ‘Sphingolipid metabolism’ and ‘Arachidonic acid metabolism’ were both impacted, being activated in B5F125 compared with B5F75 cows. However, because flux in ‘sphingolipid metabolism’ was close to zero (-0.58), we considered the pathway impacted and induced in both groups (B5F75 and B5F125). This pathway is associated with the production of ceramides, a lipid species that exerts biological effects through cellular proliferation, differentiation, and cell death, and interacts with several pathways involved in insulin resistance, oxidative stress, inflammation, and apoptosis, all of which are linked to non-alcoholic fatty liver [7].

The fact that ‘sphingolipid metabolism’ was unequivocally activated early postpartum in BCS5 cows compared with BCS4 cows, regardless of feeding management, highlights how underconditioned animals should be less susceptible to liver TAG accumulation. In a recent study, it was reported that plasma concentrations of total ceramide and monohexosylceramide increase as lactation approaches, with greater concentrations in cows exhibiting higher adiposity a month prior to parturition relative to those with a lean phenotype [8]. Furthermore, changes in plasma ceramide concentration occurred concomitantly with changes in plasma NEFA, insulin, and insulin sensitivity, leading the authors to speculate that circulating ceramides may be fundamentally involved in the homeorhetic adaptation to early lactation [8].

The activation of ‘Arachidonic acid metabolism’ in BCS5 animals fed 75% compared with 125% of requirements indicated some degree of inflammation in the former. Arachidonic acid metabolites can have both pro- and anti-inflammatory effects [9]. However, among the top DEG at 7 d postpartum with FC ≥ |3| (Additional Table 5), the pro-inflammatory pathways seem to prevail, e.g. *ALOX12* was upregulated in B5F75 cows. This enzyme catalyzes the conversion of arachidonic acid to 12-hydroxyeicosatetraenoic acid (12-HETE), a chemotactic compound in human neutrophils [10]. If biologically-active, this compound could initiate a local inflammatory response that might impair liver function. Activation of inflammatory-related hepatic functions also have been detected in feed-restricted compared with overfed cows [11]; however, despite blood biomarkers revealing greater distress in liver and a low-grade inflammatory response in feed-restricted cows prepartum, overfed cows postpartum had greater bilirubinemia and hepatic lipid accumulation leading authors to conclude that feed restriction served to “prime” the liver to better handle the postpartal metabolic and inflammatory challenges [11].

The ‘Glycerophospholipid metabolism’ pathway was impacted and activated prepartum in B5F125 compared with B5F75. One of the central metabolites of the pathway is phosphatidylcholine, the main building block of cellular membranes that are components of lipoproteins. Lecithin also is secreted in bile to increase emulsification of micelles during intestinal lipid absorption. Even if in low amounts, the greater pasture allowance prepartum in B5F125 could have led to a greater amount of lipid ingestion and the induction of phosphatidylcholine production through the ‘glycerophospholipid metabolism’ to aid in absorption and repackaging of fatty acids in the liver [12].

**REFERENCES**

1. Ji P, Osorio JS, Drackley JK, Loor JJ: **Overfeeding a moderate energy diet prepartum does not impair bovine subcutaneous adipose tissue insulin signal transduction and induces marked changes in peripartal gene network expression**. *Journal of dairy science* 2012, **95**(8):4333-4351.

2. Allen MS, Bradford BJ, Harvatine KJ: **The cow as a model to study food intake regulation**. *Annual Review of Nutrition* 2005, **25**:523-547.

3. Nakamura MT, Yudell BE, Loor JJ: **Regulation of energy metabolism by long-chain fatty acids**. *Prog Lipid Res* 2014, **53**:124-144.

4. Calder PC: **n-3 fatty acids, inflammation and immunity: new mechanisms to explain old actions**. *The Proceedings of the Nutrition Society* 2013, **72**(3):326-336.

5. Calder PC: **n-3 polyunsaturated fatty acids, inflammation, and inflammatory diseases**. *Am J Clin Nutr* 2006, **83**(6 Suppl):1505S-1519S.

6. Bertoni G, Trevisi E, Han X, Bionaz M: **Effects of inflammatory conditions on liver activity in puerperium period and consequences for performance in dairy cows**. *Journal of dairy science* 2008, **91**(9):3300-3310.

7. Pagadala M, Kasumov T, McCullough AJ, Zein NN, Kirwan JP: **Role of ceramides in nonalcoholic fatty liver disease**. *Trends Endocrinol Metab* 2012, **23**(8):365-371.

8. Rico JE, Bandaru VV, Dorskind JM, Haughey NJ, McFadden JW: **Plasma ceramides are elevated in overweight Holstein dairy cows experiencing greater lipolysis and insulin resistance during the transition from late pregnancy to early lactation**. *Journal of dairy science* 2015.

9. Samuelsson B: **Arachidonic acid metabolism: role in inflammation**. *Zeitschrift fur Rheumatologie* 1991, **50 Suppl 1**:3-6.

10. Goetzl EJ, Pickett WC: **The human PMN leukocyte chemotactic activity of complex hydroxy-eicosatetraenoic acids (HETEs)**. *J Immunol* 1980, **125**(4):1789-1791.

11. Shahzad K, Bionaz M, Trevisi E, Bertoni G, Rodriguez-Zas SL, Loor JJ: **Integrative analyses of hepatic differentially expressed genes and blood biomarkers during the peripartal period between dairy cows overfed or restricted-fed energy prepartum**. *PLoS One* 2014, **9**(6):e99757.

12. Bauchart D: **Lipid absorption and transport in ruminants**. *Journal of dairy science* 1993, **76**(12):3864-3881.
