# Supplementary material for: Prepartum body condition score and plane of nutrition affect the hepatic transcriptome during the transition period in grazing dairy cows
Source: BMC Genomics. 2016 Nov 2;17:854. doi: 10.1186/s12864-016-3191-3 (PMC5093966; doi:10.1186/s12864-016-3191-3)
Supplement: Additional file 2: Table S8. — Accession number, gene symbol and EntrezID, forward and reverse primer sequences and product length of target genes for microarray validation. (DOCX 86 kb) [file 12864_2016_3191_MOESM2_ESM.docx]

**Table S8.** Accession number, gene symbol and EntrezID, forward and reverse primer sequences and product length of target genes for microarray validation.

| **Accession #** | **EntrezID** | **Symbol** | **Forward sequence** | **Reverse Sequence** | **Lenght** |
| --- | --- | --- | --- | --- | --- |
| NM_001081581.1 | 280969 | *ABAT* | TCATGTGGTACCGGAGCAAG | GAATGGCTTTGGAGTGCGTG | 174 |
| NM_001205333.1 | 515338 | *ACACB* | CACCCGCACCGGATGAC | CCTCGAATCTCTGGAAGGCG | 139 |
| NM_001192336.1 | 407169 | *ALOX12* | AGGGCCAGCTGGACTGGTAT | CGGACATCAGGTAGTGAGCC | 121 |
| NM_001075321.1 | 507559 | *B4GALT7* | GCCCTTTCGAGAACGCTTTG | TGCCCGGTTGAACCTGAAAT | 130 |
| NM_001083644.1 | 505926 | *BCAT1* | GGCCCCACGATGAAGGATT | TAAAACGGTGGCTCGTGTGA | 123 |
| NM_001113232.2 | 514028 | *DSE* | TGGAGTACCTCCCTCCTTGG | TCACCAACCAACTAGGCTGAG | 177 |
| NM_001100337.1 | 511060 | *EXTL1* | GCACAGGAAGGCCAGTGAC | TGTGAGAAAGCCGACCATCC | 157 |
| NM_174077.4 | 281210 | *GPX3* | CCCTGCAACCAATTTGGAAA | ACATACTTGAGGGTGGCTAGGATCT | 78 |
| NM_001076814.1 | 507817 | *ME2* | GCTCGAGTCCAGCAGAGC | TTTCTCACCCCGCTTCTTGC | 179 |
| NM_001206826.1 | 535975 | *UST* | AGAACAAACGTTCCCACAGGT | TGTACCTGGGAAGGACCCAA | 113 |
| NM_001008667.1 | 493989 | *XYLT2* | GGCCGAGTCCTTCTTTCACA | CAGCTTGCGGTTCCAGTTG | 145 |
