# Supplementary material for: Prepartum body condition score and plane of nutrition affect the hepatic transcriptome during the transition period in grazing dairy cows
Source: BMC Genomics. 2016 Nov 2;17:854. doi: 10.1186/s12864-016-3191-3 (PMC5093966; doi:10.1186/s12864-016-3191-3)
Supplement: Additional file 3: Table S9. — qPCR performance of genes measured for microarray verification. (DOCX 59 kb) [file 12864_2016_3191_MOESM3_ESM.docx]

**Additional Table 9.** qPCR performance of measured genes for microarray validation.

| **Gene** | **Median Ct^1^** | **Median ∆Ct^2^** | **Slope^3^** | **(R^2^)^4^** | **Efficiency**^5^ |  |
| --- | --- | --- | --- | --- | --- | --- |
| ***ABAT*** | 21.81 | 0.69 | -3.183 | 0.996 | 2.061 |  |
| ***ACACB*** | 32.28 | 11.37 | -3.194 | 0.975 | 2.056 |  |
| ***ALOX12*** | 30.47 | 9.41 | -3.014 | 0.998 | 2.147 |  |
| ***B4GALT7*** | 25.72 | 4.72 | -3.089 | 0.998 | 2.108 |  |
| ***BCAT1*** | 29.53 | 8.58 | -3.453 | 0.976 | 1.948 |  |
| ***DSE*** | 28.15 | 7.20 | -3.043 | 0.994 | 2.131 |  |
| ***EXTL1*** | 27.25 | 6.20 | -3.036 | 0.994 | 2.135 |  |
| ***GPX3*** | 20.506 | -0.521 | -3.225 | 0.999 | 2.042 |  |
| ***ME2*** | 26.935 | 5.995 | -3.280 | 0.992 | 2.018 |  |
| ***UST*** | 24.763 | 3.700 | -3.183 | 0.990 | 2.061 |  |
| ***XYLT2*** | 25.935 | 4.936 | -3.380 | 0.994 | 1.976 |  |
| ^1^ The median is calculated considering all cows  ^2^ The median of ∆Ct is calculated as [Ct gene – geometrical mean of Ct internal controls]  ^3^ Slope of the standard curve.  ^4^ R^2^ stands for the coefficient of determination of the standard curve.  ^5^ Efficiency is calculated as [10^(-1 / Slope)^]. | | | | | | |
