# Supplementary material for: Prepartum body condition score and plane of nutrition affect the hepatic transcriptome during the transition period in grazing dairy cows
Source: BMC Genomics. 2016 Nov 2;17:854. doi: 10.1186/s12864-016-3191-3 (PMC5093966; doi:10.1186/s12864-016-3191-3)
Supplement: Additional file 4: Table S1. — Differentially expressed genes at −7 days from parturition with fold change (FC) ≤ −3 or ≥ +3 in liver of animals with BCS 4 fed either 125 (B4F125) compared with 75 (B4F75) % of requirement for the last 3 weeks before parturition. (DOCX 99 kb) [file 12864_2016_3191_MOESM4_ESM.docx]

| **Table S1.** Differentially expressed genes at -7 days from parturitionwith fold change (FC) ≤ −3 or ≥ +3 in liver of animals with BCS 4 fed either 125 (B4F125) compared with 75 (B4F75) % of requirement for the last 3 weeks before parturition. | | |
| --- | --- | --- |
| **Gene** | **Description** | **FC at -7 d** |
| ***Upregulated*** | | |
| *LOC788413* | olfactory receptor, family 10, subfamily C, member 1-like | 11,16 |
| *PTPRU* | protein tyrosine phosphatase, receptor type, U | 4,81 |
| *KIAA1045* | KIAA1045 ortholog | 4,66 |
| *OBSCN* | obscurin, cytoskeletal calmodulin and titin-interacting RhoGEF | 4,45 |
| *ARHGAP21* | Rho GTPase activating protein 21 | 4,35 |
| *AGR2* | anterior gradient homolog 2 | 4,25 |
| *ZNF445* | zinc finger protein 445 | 4,21 |
| *LOC100137795* | uncharacterized LOC100137795 | 4,20 |
| *SPATA2* | spermatogenesis associated 2 | 4,00 |
| *KIAA0319L* | KIAA0319-like ortholog | 3,87 |
| *DCP1A* | DCP1 decapping enzyme homolog A | 3,84 |
| *GTF2A1* | general transcription factor IIA, 1, 19/37kDa | 3,81 |
| *ANKRD34B* | ankyrin repeat domain 34B | 3,78 |
| *PTPN4* | protein tyrosine phosphatase, non-receptor type 4 | 3,77 |
| *RNPEP* | arginyl aminopeptidase | 3,39 |
| *PLCL2* | phospholipase C-like 2 | 3,34 |
| *BDA20* | major allergen BDA20 | 3,25 |
| *C25H16orf45* | chromosome 25 open reading frame, human C16orf45 | 3,08 |
| *MBD1* | methyl-CpG binding domain protein 1 | 3,02 |
| ***Downregulated*** | | |
| *MAU2* | MAU2 chromatid cohesion factor homolog | -7,61 |
| *ZNF236* | zinc finger protein 236 | -7,49 |
| *LRRC49* | leucine rich repeat containing 49 | -5,34 |
| *AQP6* | aquaporin 6 | -4,85 |
| *CACNB2* | calcium channel, voltage-dependent, beta 2 subunit | -4,56 |
| *LOC789904* | ribosomal protein L7a-like | -4,30 |
| *FGF11* | fibroblast growth factor 11 | -3,97 |
| *SSPO* | SCO-spondin homolog | -3,95 |
| *LRRTM3* | leucine rich repeat transmembrane neuronal 3 | -3,59 |
| *CDH7* | cadherin 7, type 2 | -3,59 |
| *LOC524560* | olfactory receptor, family 10, subfamily H, member 1-like | -3,50 |
| *SASH3* | SAM and SH3 domain containing 3 | -3,49 |
| *EZH2* | enhancer of zeste homolog 2 | -3,39 |
| *C2CD4D* | C2 calcium-dependent domain containing 4D | -3,36 |
| *KCNG3* | potassium voltage-gated channel, subfamily G, member 3 | -3,31 |
| *PTH2R* | parathyroid hormone 2 receptor | -3,19 |
| *KCTD1* | potassium channel tetramerization domain containing 1 | -3,11 |
| *HIATL1* | hippocampus abundant transcript-like 1 | -3,11 |
| *RHOF* | ras homolog gene family, member F | -3,05 |
| *PDLIM7* | PDZ and LIM domain 7 | -3,03 |
| *MS4A18* | membrane-spanning 4-domains, subfamily A, member 18 | -3,03 |
| *WDR87* | WD repeat domain 87 | -3,01 |
