# Supplementary material for: Prepartum body condition score and plane of nutrition affect the hepatic transcriptome during the transition period in grazing dairy cows
Source: BMC Genomics. 2016 Nov 2;17:854. doi: 10.1186/s12864-016-3191-3 (PMC5093966; doi:10.1186/s12864-016-3191-3)
Supplement: Additional file 5: Table S2. — Differentially expressed genes at +7 days from parturition with fold change (FC) ≤ −3 or ≥ +3 in liver of animals with BCS 4 fed either 125 (B4F125) compared with 75 (B4F75) % of requirement for the three weeks before parturition. (DOCX 114 kb) [file 12864_2016_3191_MOESM5_ESM.docx]

| **Table S2.** Differentially expressed genes at +7 days from parturitionwith fold change (FC) ≤ −3 or ≥ +3 in liver of animals with BCS 4 fed either 125 (B4F125) compared with 75 (B4F75) % of requirement for the three weeks before parturition. | | |
| --- | --- | --- |
| **Gene** | **Description** | **FC at +7 d** |
| ***Upregulated*** | | |
| *SOSTDC1* | sclerostin domain containing 1 | 12,07 |
| *MYOC* | myocilin, trabecular meshwork inducible glucocorticoid response | 7,45 |
| *MBOAT2* | membrane bound O-acyltransferase domain containing 2 | 6,94 |
| *SYT15* | synaptotagmin XV | 6,71 |
| *HSFY2* | heat shock transcription factor, Y linked 2 | 6,23 |
| *GABRA6* | gamma-aminobutyric acid (GABA) A receptor, alpha 6 | 5,69 |
| *GAD2* | glutamate decarboxylase 2 | 5,69 |
| *LOC530773* | germinal histone H4-like | 5,54 |
| *GALNT2* | UDP-N-acetyl-alpha-D-galactosamine:polypeptide N-acetylgalactosaminyltransferase 2 (GalNAc-T2) | 5,41 |
| *FSCN3* | fascin homolog 3, actin-bundling protein, testicular | 5,16 |
| *SLC24A6* | solute carrier family 24 (sodium/potassium/calcium exchanger), member 6 | 4,86 |
| *ANAPC15* | anaphase promoting complex subunit 15 | 4,83 |
| *SLC16A14* | solute carrier family 16, member 14 (monocarboxylic acid transporter 14) | 4,61 |
| *DGAT1* | diacylglycerol O-acyltransferase 1 | 4,50 |
| *HS3ST2* | heparan sulfate (glucosamine) 3-O-sulfotransferase 2 | 4,26 |
| *EIF5A* | eukaryotic translation initiation factor 5A | 4,24 |
| *AARS* | alanyl-tRNA synthetase | 4,19 |
| *GRM1* | glutamate receptor, metabotropic 1 | 4,06 |
| *CRLF2* | cytokine receptor-like factor 2 | 4,04 |
| *GOLGA1* | golgin A1 | 3,98 |
| *PALM2* | paralemmin 2 | 3,94 |
| *TRIM63* | tripartite motif containing 63 | 3,89 |
| *TCRA* | T cell receptor, alpha | 3,88 |
| *GJD4* | gap junction protein, delta 4, 40.1kDa | 3,87 |
| *HRH4* | histamine receptor H4 | 3,84 |
| *MGAT5* | mannosyl (alpha-1,6-)-glycoprotein beta-1,6-N-acetyl-glucosaminyltransferase | 3,84 |
| *MUC13* | mucin 13, cell surface associated | 3,83 |
| *NFE2L1* | nuclear factor (erythroid-derived 2)-like 1 | 3,79 |
| *MAK* | male germ cell-associated kinase | 3,74 |
| *LOC783998* | olfactory receptor, family 11, subfamily G, member 2-like | 3,69 |
| *TTLL6* | tubulin tyrosine ligase-like family, member 6 | 3,69 |
| *HERC2* | hect domain and RLD 2 | 3,68 |
| *ATG16L1* | ATG16 autophagy related 16-like 1 | 3,64 |
| *CACNA1A* | calcium channel, voltage-dependent, P/Q type, alpha 1A subunit | 3,62 |
| *TMEM160* | transmembrane protein 160 | 3,57 |
| *FAM84B* | family with sequence similarity 84, member B | 3,48 |
| *ASXL3* | additional sex combs like 3 | 3,48 |
| *CELF4* | CUGBP, Elav-like family member 4 | 3,46 |
| *LRRC73* | leucine rich repeat containing 73 | 3,43 |
| *LBH* | limb bud and heart development | 3,42 |
| *OR52W1* | olfactory receptor, family 52, subfamily W, member 1 | 3,42 |
| *TLR7* | toll-like receptor 7 | 3,41 |
| *RPRD2* | regulation of nuclear pre-mRNA domain containing 2 | 3,41 |
| *KCNIP1* | Kv channel interacting protein 1 | 3,39 |
| *SP4* | Sp4 transcription factor | 3,35 |
| *LOC615782* | PWWP domain-containing protein MUM1L1 | 3,34 |
| *TTLL8* | tubulin tyrosine ligase-like family, member 8 | 3,32 |
| *MAST1* | microtubule associated serine/threonine kinase 1 | 3,32 |
| *NKIRAS2* | NFKB inhibitor interacting Ras-like 2 | 3,31 |
| *MARCH10* | membrane-associated ring finger (C3HC4) 10, E3 ubiquitin protein ligase | 3,31 |
| *SLC14A1* | solute carrier family 14 (urea transporter), member 1 | 3,31 |
| *THBS2* | thrombospondin 2 | 3,29 |
| *ZFYVE20* | zinc finger, FYVE domain containing 20 | 3,29 |
| *PPP1R9A* | protein phosphatase 1, regulatory subunit 9A | 3,29 |
| *ENTPD6* | ectonucleoside triphosphate diphosphohydrolase 6 | 3,27 |
| *LOC614895* | olfactory receptor, family 2, subfamily T, member 27-like | 3,26 |
| *DDX4* | DEAD (Asp-Glu-Ala-Asp) box polypeptide 4 | 3,22 |
| *SLC8A1* | solute carrier family 8 (sodium/calcium exchanger), member 1 | 3,21 |
| *SLC18A2* | solute carrier family 18 (vesicular monoamine), member 2 | 3,20 |
| *WDR16* | WD repeat domain 16 | 3,19 |
| *SLC6A9* | solute carrier family 6 (neurotransmitter transporter, glycine), member 9 | 3,16 |
| *IPO4* | importin 4 | 3,15 |
| *SLC22A17* | solute carrier family 22, member 17 | 3,15 |
| *KLK4* | kallikrein-related peptidase 4 | 3,14 |
| *VWA2* | von Willebrand factor A domain containing 2 | 3,14 |
| *KCNT1* | potassium channel, subfamily T, member 1 | 3,13 |
| *ABCA13* | ATP-binding cassette, sub-family A (ABC1), member 13 | 3,13 |
| *ACTR3B* | ARP3 actin-related protein 3 homolog B | 3,12 |
| *SSTR2* | somatostatin receptor 2 | 3,10 |
| *COL4A6* | collagen, type IV, alpha 6 | 3,09 |
| *TXNDC8* | thioredoxin domain containing 8 | 3,09 |
| *MARCH4* | membrane-associated ring finger (C3HC4) 4, E3 ubiquitin protein ligase | 3,08 |
| *WC1.3* | WC1.3 molecule | 3,07 |
| *MYH9* | myosin, heavy chain 9, non-muscle | 3,06 |
| *NLN* | neurolysin (metallopeptidase M3 family) | 3,06 |
| *APLP2* | amyloid beta (A4) precursor-like protein 2 | 3,05 |
| *LOC100847827* | transcription elongation regulator 1-like protein-like | 3,05 |
| *DOPEY1* | dopey family member 1 | 3,02 |
| *ATP5SL* | ATP5S-like | 3,02 |
| *NDP* | Norrie disease | 3,01 |
| *BTN1A1* | butyrophilin, subfamily 1, member A1 | 3,01 |
| *CALM* | calmodulin-like | 3,01 |
| *TNR* | tenascin R | 3,00 |
| *LOC100125776* | olfactory receptor | 3,00 |
| ***Downregulated*** | | |
| *ASPM* | asp (abnormal spindle) homolog, microcephaly associated | -9,75 |
| *BUB1* | budding uninhibited by benzimidazoles 1 homolog | -8,29 |
| *O3FAR1* | omega-3 fatty acid receptor 1 | -7,98 |
| *HIP1* | huntingtin interacting protein 1 | -6,12 |
| *GLRA4* | glycine receptor, alpha 4 | -5,88 |
| *LGSN* | lengsin, lens protein with glutamine synthetase domain | -5,60 |
| *TMEM169* | transmembrane protein 169 | -5,50 |
| *CCK* | cholecystokinin | -5,36 |
| *GPX3* | glutathione peroxidase 3 | -5,25 |
| *INS* | insulin | -5,24 |
| *CAMKK1* | calcium/calmodulin-dependent protein kinase kinase 1, alpha | -5,18 |
| *LOC787081* | UPF0632 protein A | -5,13 |
| *KIF4A* | kinesin family member 4A | -4,88 |
| *DAB2IP* | DAB2 interacting protein | -4,69 |
| *CKAP2* | cytoskeleton associated protein 2 | -4,69 |
| *CHRDL1* | chordin-like 1 | -4,61 |
| *RNASEH2A* | ribonuclease H2, subunit A | -4,34 |
| *RRM2* | ribonucleotide reductase M2 | -4,34 |
| *GRASP* | GRP1 (general receptor for phosphoinositides 1)-associated scaffold protein | -4,22 |
| *LOC526064* | histone cluster 1, H2bl-like | -4,13 |
| *PLIN4* | perilipin 4 | -4,07 |
| *RNF168* | ring finger protein 168, E3 ubiquitin protein ligase | -4,01 |
| *LOC539789* | stathmin-like pseudogene | -4,01 |
| *MC4R* | melanocortin 4 receptor | -3,75 |
| *LOC524985* | olfactory receptor 1038-like | -3,71 |
| *ABCC9* | ATP-binding cassette, sub-family C (CFTR/MRP), member 9 | -3,71 |
| *OVOL2* | ovo-like 2 (Drosophila) | -3,66 |
| *AGR2* | anterior gradient homolog 2 | -3,56 |
| *MEIS2* | Meis homeobox 2 | -3,53 |
| *E2F1* | E2F transcription factor 1 | -3,52 |
| *NKG2C* | NKG2C protein | -3,49 |
| *UBE2C* | ubiquitin-conjugating enzyme E2C | -3,48 |
| *MYBL2* | v-myb avian myeloblastosis viral oncogene homolog-like 2 | -3,47 |
| *ME1* | malic enzyme 1, NADP(+)-dependent, cytosolic | -3,45 |
| *GPNMB* | glycoprotein (transmembrane) nmb | -3,34 |
| *BAALC* | brain and acute leukemia, cytoplasmic | -3,33 |
| *MEF2C* | myocyte enhancer factor 2C | -3,33 |
| *LOC787642* | Olfactory receptor-like protein OLF2-like | -3,28 |
| *LOC510902* | olfactory receptor, family 4, subfamily B, member 1-like | -3,27 |
| *CCDC162P* | coiled-coil domain containing 162, pseudogene | -3,26 |
| *VLDLR* | very low density lipoprotein receptor | -3,26 |
| *MED13L* | mediator complex subunit 13-like | -3,25 |
| *MAPRE3* | microtubule-associated protein, RP/EB family, member 3 | -3,25 |
| *COL8A1* | collagen, type VIII, alpha 1 | -3,21 |
| *LRRC16A* | leucine rich repeat containing 16A | -3,20 |
| *WDR93* | WD repeat domain 93 | -3,18 |
| *VILL* | villin-like | -3,17 |
| *TMEM38A* | transmembrane protein 38A | -3,16 |
| *IKZF1* | IKAROS family zinc finger 1 (Ikaros) | -3,15 |
| *C2H1orf64* | chromosome 2 open reading frame, human C1orf64 | -3,12 |
| *METTL19* | methyltransferase like 19 | -3,09 |
| *FAM13C* | family with sequence similarity 13, member C | -3,08 |
| *RFX5* | regulatory factor X, 5 (influences HLA class II expression) | -3,08 |
| *RGS17* | regulator of G-protein signaling 17 | -3,07 |
| *KCNMB1* | potassium large conductance calcium-activated channel, subfamily M, beta member 1 | -3,07 |
| *MARS* | methionyl-tRNA synthetase | -3,05 |
| *SPC24* | SPC24, NDC80 kinetochore complex component, homolog | -3,05 |
| *WT1* | Wilms tumor 1 | -3,04 |
| *LOC789904* | ribosomal protein L7a-like | -3,04 |
| *BOLA-DMB* | major histocompatibility complex, class II, DMB | -3,02 |
| *LOC618591* | killer cell lectin-like receptor-like | -3,02 |
