# Supplementary material for: Prepartum body condition score and plane of nutrition affect the hepatic transcriptome during the transition period in grazing dairy cows
Source: BMC Genomics. 2016 Nov 2;17:854. doi: 10.1186/s12864-016-3191-3 (PMC5093966; doi:10.1186/s12864-016-3191-3)
Supplement: Additional file 6: Table S3. — Differentially expressed genes at +28 days from parturition with fold change (FC) ≤ −3 or ≥ +3 in liver of animals with BCS 4 fed either 125 (B4F125) compared with 75 (B4F75) % of requirement for the three weeks before parturition. (DOCX 110 kb) [file 12864_2016_3191_MOESM6_ESM.docx]

| **Table S3.** Differentially expressed genes at +28 days from parturition with fold change (FC) ≤ −3 or ≥ +3 in liver of animals with BCS 4 fed either 125 (B4F125) compared with 75 (B4F75) % of requirement for the three weeks before parturition. | | |
| --- | --- | --- |
| **Gene** | **Description** | **FC at +28 d** |
| ***Upregulated*** | | |
| *HSPA1A* | heat shock 70kDa protein 1A | 4,27 |
| *MYO7B* | myosin VIIB | 3,82 |
| *MMP9* | matrix metallopeptidase 9 (gelatinase B, 92kDa gelatinase, 92kDa type IV collagenase) | 3,69 |
| *PPP1R21* | protein phosphatase 1, regulatory subunit 21 | 3,41 |
| *CDHR1* | cadherin-related family member 1 | 3,31 |
| *ALPK1* | alpha-kinase 1 | 3,26 |
| *APOA4* | apolipoprotein A-IV | 3,25 |
| *FGF6* | fibroblast growth factor 6 | 3,19 |
| ***Downregulated*** |  |  |
| *CD46* | CD46 molecule, complement regulatory protein | -9,87 |
| *KRT9* | keratin 9 | -9,84 |
| *PPP1R14C* | protein phosphatase 1, regulatory (inhibitor) subunit 14C | -8,70 |
| *AKAP6* | A kinase (PRKA) anchor protein 6 | -8,29 |
| *KIR2DL5A* | killer cell immunoglobulin-like receptor, two domains, long cytoplasmic tail, 5A | -7,86 |
| *FGF8* | fibroblast growth factor 8 (androgen-induced) | -7,40 |
| *LOC100139191* | uncharacterized LOC100139191 | -7,36 |
| *FGD3* | FYVE, RhoGEF and PH domain containing 3 | -7,33 |
| *C7H5orf46* | chromosome 7 open reading frame, human C5orf46 | -7,22 |
| *SGIP1* | SH3-domain GRB2-like (endophilin) interacting protein 1 | -7,21 |
| *TCHHL1* | trichohyalin-like 1 | -6,97 |
| *PLEKHG7* | pleckstrin homology domain containing, family G (with RhoGef domain) member 7 | -6,85 |
| *MYPN* | myopalladin | -6,18 |
| *OPN1SW* | opsin 1 (cone pigments), short-wave-sensitive | -6,05 |
| *LOC789829* | sorting nexin-5-like | -6,01 |
| *RYR1* | ryanodine receptor 1 | -5,84 |
| *KIAA1456* | putative methyltransferase KIAA1456 homolog | -5,79 |
| *OVCH2* | ovochymase 2 | -5,78 |
| *AGR2* | anterior gradient homolog 2 | -5,34 |
| *LALBA* | lactalbumin, alpha- | -5,14 |
| *SLC35D3* | solute carrier family 35, member D3 | -5,10 |
| *C26H10orf90* | chromosome 26 open reading frame, human C10orf90 | -5,08 |
| *ALPK3* | alpha-kinase 3 | -5,06 |
| *KRT6B* | keratin 6B | -5,04 |
| *CCDC178* | coiled-coil domain containing 178 | -5,01 |
| *METTL24* | methyltransferase like 24 | -4,95 |
| *LOC786089* | establishment of cohesion 1 homolog 2-like | -4,93 |
| *TGM7* | transglutaminase 7 | -4,91 |
| *PPP1R3A* | protein phosphatase 1, regulatory subunit 3A | -4,90 |
| *NEUROD6* | neurogenic differentiation 6 | -4,87 |
| *SVOP* | SV2 related protein homolog (rat) | -4,85 |
| *CHRDL1* | chordin-like 1 | -4,78 |
| *NIM1* | serine/threonine-protein kinase NIM1 | -4,73 |
| *MMP13* | matrix metallopeptidase 13 (collagenase 3) | -4,64 |
| *LOC532848* | centrosomal protein KIAA1731-like | -4,61 |
| *RYR2* | ryanodine receptor 2 (cardiac) | -4,58 |
| *HDAC9* | histone deacetylase 9 | -4,53 |
| *ACHE* | acetylcholinesterase | -4,52 |
| *LAT2* | linker for activation of T cells family, member 2 | -4,47 |
| *CTLA4* | cytotoxic T-lymphocyte-associated protein 4 | -4,45 |
| *GABRA1* | gamma-aminobutyric acid (GABA) A receptor, alpha 1 | -4,44 |
| *TAF1C* | TATA box binding protein (TBP)-associated factor, RNA polymerase I, C, 110kDa | -4,35 |
| *MCF2* | MCF.2 cell line derived transforming sequence | -4,34 |
| *GLYCAM1* | glycosylation-dependent cell adhesion molecule 1 | -4,33 |
| *GHRL* | ghrelin/obestatin prepropeptide | -4,33 |
| *MTMR12* | myotubularin related protein 12 | -4,29 |
| *CD4* | CD4 molecule | -4,28 |
| *TEX13B* | testis expressed 13B | -4,27 |
| *DLGAP5* | discs, large (Drosophila) homolog-associated protein 5 | -4,24 |
| *LOC618010* | olfactory receptor, family 51, subfamily F, member 2-like | -4,22 |
| *GPR77* | G protein-coupled receptor 77 | -4,20 |
| *LOC508589* | olfactory receptor, family 8, subfamily A, member 1-like | -4,20 |
| *PI3* | peptidase inhibitor 3, skin-derived (SKALP) | -4,19 |
| *IKZF1* | IKAROS family zinc finger 1 (Ikaros) | -4,11 |
| *ATP2B3* | ATPase, Ca++ transporting, plasma membrane 3 | -4,09 |
| *ITGA4* | integrin, alpha 4 (antigen CD49D, alpha 4 subunit of VLA-4 receptor) | -4,08 |
| *UNC5B* | unc-5 homolog B | -4,07 |
| *NKX3-1* | NK3 homeobox 1 | -4,04 |
| *DNAI2* | dynein, axonemal, intermediate chain 2 | -4,02 |
| *PLAU* | plasminogen activator, urokinase | -4,02 |
| *KRT20* | keratin 20 | -4,02 |
| *ATP8A2* | ATPase, aminophospholipid transporter, class I, type 8A, member 2 | -4,02 |
| *DYNLRB1* | dynein, light chain, roadblock-type 1 | -4,02 |
| *GPR88* | G protein-coupled receptor 88 | -4,00 |
| *SLX4* | SLX4 structure-specific endonuclease subunit homolog | -3,98 |
| *GALNT13* | UDP-N-acetyl-alpha-D-galactosamine:polypeptide N-acetylgalactosaminyltransferase 13 (GalNAc-T13) | -3,97 |
| *SUV39H1* | suppressor of variegation 3-9 homolog 1 | -3,96 |
| *TUBB1* | tubulin, beta 1 class VI | -3,95 |
| *VTCN1* | V-set domain containing T cell activation inhibitor 1 | -3,92 |
| *C1H3orf52* | chromosome 1 open reading frame, human C3orf52 | -3,91 |
| *PSMF1* | proteasome (prosome, macropain) inhibitor subunit 1 (PI31) | -3,90 |
| *SMIM24* | small integral membrane protein 24 | -3,87 |
| *GPNMB* | glycoprotein (transmembrane) nmb | -3,85 |
| *PCDH10* | protocadherin 10 | -3,82 |
| *SYMPK* | symplekin | -3,77 |
| *LOC785406* | olfactory receptor 5AL1 | -3,74 |
| *KIF5C* | kinesin family member 5C | -3,74 |
| *RNF183* | ring finger protein 183 | -3,72 |
| *LOC508124* | homer protein homolog 3-like | -3,71 |
| *LOC506891* | olfactory receptor, family 9, subfamily G, member 4-like | -3,71 |
| *PTPN22* | protein tyrosine phosphatase, non-receptor type 22 (lymphoid) | -3,67 |
| *NR4A2* | nuclear receptor subfamily 4, group A, member 2 | -3,67 |
| *CNNM1* | cyclin M1 | -3,66 |
| *GINS2* | GINS complex subunit 2 (Psf2 homolog) | -3,65 |
| *RAX2* | retina and anterior neural fold homeobox 2 | -3,64 |
| *NXF3* | nuclear RNA export factor 3 | -3,64 |
| *IQGAP3* | IQ motif containing GTPase activating protein 3 | -3,62 |
| *SPATS1* | spermatogenesis associated, serine-rich 1 | -3,61 |
| *FGF18* | fibroblast growth factor 18 | -3,60 |
| *PTX3* | pentraxin 3, long | -3,58 |
| *SCN1A* | sodium channel, voltage-gated, type I, alpha subunit | -3,55 |
| *RBM28* | RNA binding motif protein 28 | -3,54 |
| *CDH8* | cadherin 8, type 2 | -3,54 |
| *SCRN1* | secernin 1 | -3,51 |
| *LOC523060* | olfactory receptor, family 4, subfamily A, member 15-like | -3,49 |
| *FAM70A* | family with sequence similarity 70, member A | -3,46 |
| *PRKCD* | protein kinase C, delta | -3,45 |
| *PREX1* | phosphatidylinositol-3,4,5-trisphosphate-dependent Rac exchange factor 1 | -3,44 |
| *SLC46A2* | solute carrier family 46, member 2 | -3,44 |
| *SAA3* | serum amyloid A 3 | -3,42 |
| *DPYSL4* | dihydropyrimidinase-like 4 | -3,40 |
| *LOC618422* | huntingtin-associated protein 1-like | -3,38 |
| *RPS29* | ribosomal protein S29 | -3,38 |
| *OLFM4* | olfactomedin 4 | -3,36 |
| *F2RL1* | coagulation factor II (thrombin) receptor-like 1 | -3,35 |
| *PADI4* | peptidyl arginine deiminase, type IV | -3,32 |
| *CHRAC1* | chromatin accessibility complex 1 | -3,31 |
| *CLEC4E* | C-type lectin domain family 4, member E | -3,30 |
| *SHH* | sonic hedgehog | -3,29 |
| *TNFRSF9* | tumor necrosis factor receptor superfamily, member 9 | -3,26 |
| *TAP* | tracheal antimicrobial peptide | -3,25 |
| *RPRD2* | regulation of nuclear pre-mRNA domain containing 2 | -3,24 |
| *CCDC60* | coiled-coil domain containing 60 | -3,23 |
| *ARMC3* | armadillo repeat containing 3 | -3,23 |
| *ARF1* | ADP-ribosylation factor 1 | -3,23 |
| *FLOT2* | flotillin 2 | -3,22 |
| *TKDP2* | trophoblast Kunitz domain protein 2 | -3,22 |
| *LOC510902* | olfactory receptor, family 4, subfamily B, member 1-like | -3,21 |
| *CLSPN* | claspin | -3,18 |
| *LYZ1* | ysozyme 1 | -3,17 |
| *GDPD3* | glycerophosphodiester phosphodiesterase domain containing 3 | -3,15 |
| *DLX4* | distal-less homeobox 4 | -3,15 |
| *C14H8orf34* | chromosome 14 open reading frame, human C8orf34 | -3,15 |
| *PTPRU* | protein tyrosine phosphatase, receptor type, U | -3,14 |
| *FRY* | furry homolog | -3,14 |
| *BOSTAUV1R404* | vomeronasal 1 receptor bosTauV1R404 | -3,13 |
| *CCDC136* | coiled-coil domain containing 136 | -3,12 |
| *INTU* | inturned planar cell polarity effector homolog | -3,12 |
| *SNRPC* | small nuclear ribonucleoprotein polypeptide C | -3,11 |
| *ZZEF1* | zinc finger, ZZ-type with EF-hand domain 1 | -3,10 |
| *VAV1* | vav 1 guanine nucleotide exchange factor | -3,10 |
| *YBX2* | Y box binding protein 2 | -3,09 |
| *CLOCK* | clock circadian regulator | -3,09 |
| *OR6C75* | olfactory receptor, family 6, subfamily C, member 75 | -3,08 |
| *ZNF383* | zinc finger protein 383 | -3,06 |
| *TK1* | thymidine kinase 1, soluble | -3,06 |
| *LOC532330* | insulin receptor substrate 3-like | -3,04 |
| *RCOR1* | REST corepressor 1 | -3,04 |
| *RASAL2* | RAS protein activator like 2 | -3,03 |
| *SNX29* | sorting nexin 29 | -3,03 |
| *DYTN* | dystrotelin | -3,01 |
| *NTS* | neurotensin | -3,00 |
