# Supplementary material for: Prepartum body condition score and plane of nutrition affect the hepatic transcriptome during the transition period in grazing dairy cows
Source: BMC Genomics. 2016 Nov 2;17:854. doi: 10.1186/s12864-016-3191-3 (PMC5093966; doi:10.1186/s12864-016-3191-3)
Supplement: Additional file 7: Table S4. — Differentially expressed genes at −7 days from parturition with fold change (FC) ≤ −3 or ≥ +3 in liver of animals with BCS 5 fed either 125 (B5F125) compared with 75 (B5F75) % of requirement for the three weeks before parturition. (DOCX 77 kb) [file 12864_2016_3191_MOESM7_ESM.docx]

| **Table S4.** Differentially expressed genes at -7 days from parturitionwith fold change (FC) ≤ −3 or ≥ +3 in liver of animals with BCS 5 fed either 125 (B5F125) compared with 75 (B5F75) % of requirement for the three weeks before parturition. | | | |
| --- | --- | --- | --- |
| **Gene** | **Description** | **FC at -7 d** |  |
| ***Upregulated*** | | | |
| *ESYT2* | extended synaptotagmin-like protein 2 | 5,17 |  |
| *CRYGD* | crystallin, gamma D | 4,98 |  |
| *RSPH1* | radial spoke head 1 homolog | 4,54 |  |
| *ANGPTL7* | angiopoietin-like 7 | 4,39 |  |
| *PLD5* | phospholipase D family, member 5 | 3,64 |  |
| *RHBDL2* | rhomboid, veinlet-like 2 | 3,54 |  |
| *OBSCN* | obscurin, cytoskeletal calmodulin and titin-interacting RhoGEF | 3,18 |  |
| *LOC787878* | putative olfactory receptor 56B2-like | 3,10 |  |
| ***Downregulated*** | | | |
| *C1QL3* | complement component 1, q subcomponent-like 3 | -6,66 |  |
| *CADPS* | Ca^++^-dependent secretion activator | -6,15 |  |
| *TRIM36* | tripartite motif containing 36 | -5,52 |  |
| *MAEL* | maelstrom homolog | -5,45 |  |
| *NRCAM* | neuronal cell adhesion molecule | -4,13 |  |
| *FEZF1* | FEZ family zinc finger 1 | -4,03 |  |
| *SLC26A8* | solute carrier family 26, member 8 | -3,80 |  |
| *CKMT1B* | creatine kinase, mitochondrial 1B | -3,71 |  |
| *LOC782400* | uncharacterized LOC782400 | -3,71 |  |
| *ELN* | elastin | -3,25 |  |
| *RPRD2* | regulation of nuclear pre-mRNA domain containing 2 | -3,11 |  |
| *FLNA* | filamin A, alpha | -3,09 |  |
| *RPS28* | ribosomal protein S28 | -3,08 |  |
| *FGFR2* | fibroblast growth factor receptor 2 | -3,03 |  |
