# Supplementary material for: Prepartum body condition score and plane of nutrition affect the hepatic transcriptome during the transition period in grazing dairy cows
Source: BMC Genomics. 2016 Nov 2;17:854. doi: 10.1186/s12864-016-3191-3 (PMC5093966; doi:10.1186/s12864-016-3191-3)
Supplement: Additional file 8: Table S5. — Differentially expressed genes at +7 days from parturition with fold change (FC) ≤ −3 or ≥ +3 in liver of animals with BCS 5 fed either 125 (B5F125) compared with 75 (B5F75) % of requirement for the three weeks before parturition. (DOCX 105 kb) [file 12864_2016_3191_MOESM8_ESM.docx]

| **Table S5.** Differentially expressed genes at +7 days from parturitionwith fold change (FC) ≤ −3 or ≥ +3 in liver of animals with BCS 5 fed either 125 (B5F125) compared with 75 (B5F75) % of requirement for the three weeks before parturition. | | |
| --- | --- | --- |
| **Gene** | **Description** | **FC at +7 d** |
| ***Upregulated*** | | |
| *CRB1* | crumbs homolog 1 | 7,77 |
| *ALPK1* | alpha-kinase 1 | 5,87 |
| *TMEM163* | transmembrane protein 163 | 5,18 |
| *ARL14* | ADP-ribosylation factor-like 14 | 4,76 |
| *DAB2IP* | DAB2 interacting protein | 4,66 |
| *IPPK* | inositol 1,3,4,5,6-pentakisphosphate 2-kinase | 4,65 |
| *CALML5* | calmodulin-like 5 | 4,41 |
| *LOC616722* | immunity-related GTPase family, cinema-like | 4,30 |
| *LOC618985* | interferon, omega 1-like | 4,16 |
| *XPO4* | exportin 4 | 4,15 |
| *PGPEP1L* | pyroglutamyl-peptidase I-like | 4,00 |
| *LOC539818* | ribophorin I-like | 3,97 |
| *MCART6* | mitochondrial carrier triple repeat 6 | 3,93 |
| *RASD2* | RASD family, member 2 | 3,79 |
| *MAN2C1* | mannosidase, alpha, class 2C, member 1 | 3,73 |
| *LOC788761* | uncharacterized LOC788761 | 3,72 |
| *C10H14orf39* | chromosome 10 open reading frame, human C14orf39 | 3,69 |
| *CCK* | cholecystokinin | 3,66 |
| *MAP3K12* | mitogen-activated protein kinase kinase kinase 12 | 3,66 |
| *NEIL3* | nei endonuclease VIII-like 3 | 3,57 |
| *LOC509124* | olfactory receptor, family 9, subfamily G, member 4-like | 3,51 |
| *BMP7* | bone morphogenetic protein 7 | 3,49 |
| *CCDC113* | coiled-coil domain containing 113 | 3,47 |
| *LOC100140382* | olfactory receptor, family 4, subfamily F, member 21-like | 3,46 |
| *DBIL5* | endozepine-like peptide 2 | 3,41 |
| *LOC618124* | olfactory receptor, family 1, subfamily E, member 2-like | 3,40 |
| *CCBP2* | chemokine binding protein 2 | 3,39 |
| *LOC785309* | T-cell receptor alpha chain V region PY14-like | 3,31 |
| *TRPC4* | transient receptor potential cation channel, subfamily C, member 4 | 3,31 |
| *DNAL4* | dynein, axonemal, light chain 4 | 3,30 |
| *KCNMB2* | potassium large conductance calcium-activated channel, subfamily M, beta member 2 | 3,30 |
| *CRY1* | cryptochrome 1 (photolyase-like) | 3,29 |
| *CLDN22* | claudin 22 | 3,25 |
| *LYG2* | lysozyme G-like 2 | 3,23 |
| *STAT5A* | signal transducer and activator of transcription 5A | 3,15 |
| *PDE10A* | phosphodiesterase 10A | 3,13 |
| *LOC532330* | insulin receptor substrate 3-like | 3,13 |
| *FCGBP* | Fc fragment of IgG binding protein | 3,11 |
| *SLC6A6* | solute carrier family 6 (neurotransmitter transporter, taurine), member 6 | 3,09 |
| *LOC506670* | solute carrier family 7 (anionic amino acid transporter), member 13-like | 3,09 |
| *LOC510100* | olfactory receptor, family 10, subfamily A, member 5-like | 3,08 |
| *NKD1* | naked cuticle homolog 1 | 3,04 |
| ***Downregulated*** |  |  |
| *FIGLA* | folliculogenesis specific basic helix-loop-helix | -7,19 |
| *KCNH6* | potassium voltage-gated channel, subfamily H (eag-related), member 6 | -6,74 |
| *NDNF* | neuron-derived neurotrophic factor | -6,71 |
| *ELOVL7* | ELOVL fatty acid elongase 7 | -6,39 |
| *TTF2* | transcription termination factor, RNA polymerase II | -5,68 |
| *RTP3* | receptor (chemosensory) transporter protein 3 | -5,42 |
| *PLEKHB2* | pleckstrin homology domain containing, family B (evectins) member 2 | -5,20 |
| *PLD5* | phospholipase D family, member 5 | -4,79 |
| *IRG1* | immunoresponsive 1 homolog | -4,70 |
| *SLIT3* | slit homolog 3 | -4,50 |
| *CAPN3* | calpain 3, (p94) | -4,49 |
| *SUN3* | Sad1 and UNC84 domain containing 3 | -4,49 |
| *FGF8* | fibroblast growth factor 8 (androgen-induced) | -4,29 |
| *RS1* | retinoschisin 1 | -4,28 |
| *OR10K1* | olfactory receptor, family 10, subfamily K, member 1 | -4,18 |
| *GTPBP4* | GTP binding protein 4 | -4,10 |
| *ANXA8L1* | annexin A8-like 1 | -4,09 |
| *TRAF5* | TNF receptor-associated factor 5 | -4,02 |
| *IFI47* | interferon gamma inducible protein 47 | -3,91 |
| *UHRF1* | ubiquitin-like with PHD and ring finger domains 1 | -3,79 |
| *KRT73* | keratin 73 | -3,71 |
| *MTMR12* | myotubularin related protein 12 | -3,69 |
| *NXPH3* | neurexophilin 3 | -3,63 |
| *EFHB* | EF-hand domain family, member B | -3,61 |
| *AGAP2* | ArfGAP with GTPase domain, ankyrin repeat and PH domain 2 | -3,51 |
| *IFNA16* | interferon, alpha 16 | -3,47 |
| *TAP1* | transporter 1, ATP-binding cassette, sub-family B (MDR/TAP) | -3,46 |
| *KCNS1* | potassium voltage-gated channel, delayed-rectifier, subfamily S, member 1 | -3,32 |
| *AMOTL2* | angiomotin like 2 | -3,30 |
| *TRPA1* | transient receptor potential cation channel, subfamily A, member 1 | -3,29 |
| *ALOX12* | arachidonate 12-lipoxygenase | -3,26 |
| *FLT1* | fms-related tyrosine kinase 1 | -3,24 |
| *KCTD6* | potassium channel tetramerisation domain containing 6 | -3,21 |
| *RNF168* | ring finger protein 168, E3 ubiquitin protein ligase | -3,21 |
| *MIS18BP1* | MIS18 binding protein 1 | -3,19 |
| *NEU4* | sialidase 4 | -3,13 |
| *ADAMTS4* | ADAM metallopeptidase with thrombospondin type 1 motif, 4 | -3,13 |
| *DAB2* | disabled homolog 2, mitogen-responsive phosphoprotein | -3,12 |
| *RNF145* | ring finger protein 145 | -3,11 |
| *LRRC3B* | leucine rich repeat containing 3B | -3,10 |
| *ICOS* | inducible T-cell co-stimulator | -3,08 |
| *ME1* | malic enzyme 1, NADP(+)-dependent, cytosolic | -3,04 |
| *TMEM196* | transmembrane protein 196 | -3,01 |
| *BCAT1* | branched chain amino-acid transaminase 1, cytosolic | -3,01 |
