# Supplementary material for: Prepartum body condition score and plane of nutrition affect the hepatic transcriptome during the transition period in grazing dairy cows
Source: BMC Genomics. 2016 Nov 2;17:854. doi: 10.1186/s12864-016-3191-3 (PMC5093966; doi:10.1186/s12864-016-3191-3)
Supplement: Additional file 9: Table S6. — Differentially expressed genes at +28 days from parturition with fold change (FC) ≤ −3 or ≥ +3 in liver of animals with BCS 5 fed either 125 (B5F125) compared with 75 (B5F75) % of requirement for the three weeks before parturition. (DOCX 70 kb) [file 12864_2016_3191_MOESM9_ESM.docx]

| **Table S6.** Differentially expressed genes at +28 days from parturitionwith fold change (FC) ≤ −3 or ≥ +3 in liver of animals with BCS 5 fed either 125 (B5F125) compared with 75 (B5F75) % of requirement for the three weeks before parturition. | | | |
| --- | --- | --- | --- |
| **Gene** | **Description** | **FC at +28 d** |  |
| ***Upregulated*** | | | |
| *OPA3* | optic atrophy 3 | 6,18 |  |
| *LRRC49* | leucine rich repeat containing 49 | 5,45 |  |
| *FOXJ1* | forkhead box J1 | 3,63 |  |
| *ZNF19* | zinc finger protein 19 | 3,61 |  |
| *OTOL1* | otolin 1 | 3,59 |  |
| *CXCL11* | chemokine (C-X-C motif) ligand 11 | 3,49 |  |
| *UCN* | urocortin | 3,39 |  |
| *ZBTB46* | zinc finger and BTB domain containing 46 | 3,03 |  |
| *TNFAIP8L3* | tumor necrosis factor, alpha-induced protein 8-like 3 | 3,01 |  |
| ***Downregulated*** | | | |
| *LOC785406* | olfactory receptor 5AL1 | -6,05 |  |
| *TMEM196* | transmembrane protein 196 | -4,22 |  |
| *IFIT1* | interferon-induced protein with tetratricopeptide repeats 1 | -4,12 |  |
| *TMEM54* | transmembrane protein 54 | -3,70 |  |
| *SAA3* | serum amyloid A 3 | -3,43 |  |
| *APOBEC3A* | apolipoprotein B mRNA editing enzyme, catalytic polypeptide-like 3A | -3,42 |  |
| *M-SAA3.2* | mammary serum amyloid A3.2 | -3,34 |  |
| *MVK* | mevalonate kinase | -3,07 |  |
| *ATAD1* | ATPase family, AAA domain containing 1 | -3,03 |  |
