# Supplementary figures and images for: Prepartum body condition score and plane of nutrition affect the hepatic transcriptome during the transition period in grazing dairy cows
Source: BMC Genomics. 2016 Nov 2;17:854. doi: 10.1186/s12864-016-3191-3 (PMC5093966; doi:10.1186/s12864-016-3191-3)

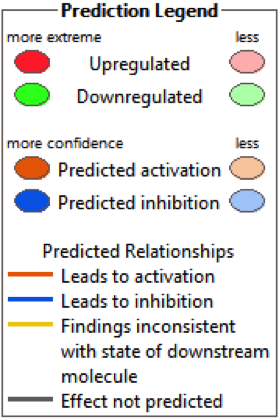

Supplement: Additional file 11: Figure S1. — Ingenuity pathway analysis legend for network analysis. (PNG 65 kb) [file 12864_2016_3191_MOESM11_ESM.png]
